# Nomenclature for Factors of the HLA System, Update July, August and September 2024

**DOI:** 10.1111/tan.15732

**Published:** 2024-11-05

**Authors:** Steven G. E. Marsh, WHO Nomenclature Committee for Factors of the HLA System

**Affiliations:** ^1^ Anthony Nolan Research Institute Royal Free Hospital London UK

The following sequences have been submitted to the Nomenclature Committee since the April, May and June 2024 nomenclature update [1] and, following agreed policy, have been assigned official allele designations [2]. Full details of all sequences will be published in a forthcoming report.

Below are listed the newly assigned sequences (Table 1) and confirmations of previously reported sequences (Table 2). The accession number of each sequence is given and these can be used to retrieve the sequence files from the EMBL, GenBank or DDBJ data libraries. Although accession numbers have been assigned by the data‐libraries and most sequences are already available, there is still the possibility that an author may not yet have allowed the sequence to be released; in such a case you will have to contact the submitting author directly. Additional information pertaining to new sequences is often included in the publications describing these alleles; a listing of recent publications that describe new HLA sequences is given in Table 3. An additional 221 alleles were recently published but have not been included in Table 3 due to space considerations [3, 4].

**TABLE 1:** New sequences.

HLA allele	Cell identification	Accession number	Submitting author
A*01:01:01:120	509860	PQ041960	Mr Alessandro Pirri, Curitiba, Brazil
A*01:464	HG00052201	PP947514	Histogenetics, Ossining, USA
A*01:465	HG00052196	PP926148	Histogenetics, Ossining, USA
A*01:466	HG00052198	PP947511	Histogenetics, Ossining, USA
A*01:467	KSR188867	PP997985	Dr Maria Loginova, Kirov, Russia
A*01:468	RP‐282626	PQ015388	Dr Neifi Hassan Saloum Deghaide, Ribeirao Preto, Brazil
A*01:469	KSR158457	PQ168561	Dr Maria Loginova, Kirov, Russia
A*01:470	2311107122	PP854397	Dr Jonathan Visentin, Bordeaux, France
A*02:01:01:256	DKMS‐LSL_ID20277195	OZ111006	DKMS Life Sciences Lab., Dresden, Germany
A*02:01:224	HG00052194	PP904454	Histogenetics, Ossining, USA
A*02:01:225	KSR192968	PQ168562	Dr Maria Loginova, Kirov, Russia
A*02:05:14	2409764	PP910681	Dr Jose Samuel da Silva, Aparecida de Goiania Goias, Brazil
A*02:1163:02	DKMS‐LSL_ID20213554	OZ111004	DKMS Life Sciences Lab., Dresden, Germany
A*02:1182	AND24630000627	PP882825	Dr Govindarajan Srinivasaraman, Chennai, India
A*02:1183	HG00052197	PP926149	Histogenetics, Ossining, USA
A*02:1184N	HG00052193	PP904453	Histogenetics, Ossining, USA
A*02:1185N	GR‐EVA‐58922	PP936197	Dr Diamanto Kouniaki, Athens, Greece
A*02:1186	9000623544635092	PP976122	Prof Faming Zhu, Hangzhou, China
A*02:1187N	108992	PP994769	Dr Celso Gouvea, Fortaleza, Brazil
A*02:1188	KSR173141	PP997983	Dr Maria Loginova, Kirov, Russia
A*02:1189	2401804	PP986396	Dr Paul Rouzaire, Clermont‐Ferrand, France
A*02:1190	DKMS‐LSL_ID19991772	OZ110994	DKMS Life Sciences Lab., Dresden, Germany
A*02:1191N	DKMS‐LSL_ID20228388	OZ110991	DKMS Life Sciences Lab., Dresden, Germany
A*02:1192	81345795	PQ124118	Dr Daniel Fuerst, Ulm, Germany
A*02:1193	KSR113794	PQ168563	Dr Maria Loginova, Kirov, Russia
A*02:1194	24N3	PQ246269	Dr Romain Ferru‐Clement, Poitiers, France
A*03:01:01:121	23‐2571	PP869412	Dr M Rosa Moya‐Quiles, El Palmar, Spain
A*03:01:01:122	201‐00511	PP978908	Miss Yue Han, Xi'an, China
A*03:01:130	2410266229	PP854399	Dr Jonathan Visentin, Bordeaux, France
A*03:01:131	DKMS‐LSL_ID20099219	OZ111001	DKMS Life Sciences Lab., Dresden, Germany
A*03:01:132	DKMS‐LSL_ID20700127	OZ111013	DKMS Life Sciences Lab., Dresden, Germany
A*03:494N	HG00052199	PP947512	Histogenetics, Ossining, USA
A*03:495	FSIMM_IMM‐24‐27987	OZ077755	Clinical Immunology, PathWest, Perth, Australia
A*03:496	DKMS‐LSL_ID20083689	OZ111000	DKMS Life Sciences Lab., Dresden, Germany
A*03:497	KSR118964	PQ168564	Dr Maria Loginova, Kirov, Russia
A*11:01:01:92	KWO010 (KAPA)	OZ121139	Anthony Nolan Research Institute, London, United Kingdom
A*11:01:132	20QB005387	OY998276	Dr Janette Kwok, Pokfulam Road, Hong Kong
A*11:01:133	136043259	PQ014358	Dr Pascal Pedini, Marseille, France
A*11:478	901047805	PP852889	Mr Thomas Vaudois, Strasbourg, Frances
A*11:479N	470067	PP953613	Prof Edward KL Yang, Hualien, Taiwan
A*11:480	21QB004586	OZ022568	Dr Janette Kwok, Pokfulam Road, Hong Kong
A*23:144	71734	PP900177	Dr Georgia Daniela Marcusso, Porto Velho, Brazil
A*23:145	HG00052200	PP947513	Histogenetics, Ossining, USA
A*23:146	900612390083526	PP976123	Prof Faming Zhu, Hangzhou, China
A*23:147	134262379	PQ014359	Dr Pascal Pedini, Marseille, France
A*24:02:01:144Q	incabrp0351	PP992535	Mr Vinicius Stelet, Rio de Janeiro, Brazil
A*24:02:176	21QB009069	OZ022573	Dr Janette Kwok, Pokfulam Road, Hong Kong
A*24:635	G231212021	OZ059285	Miss Xingjie Li, Chengdu, China
A*24:636N	HG00052195	PP904455	Histogenetics, Ossining, USA
A*24:637	FSIMM_IMM‐20‐20680	OZ077737	Clinical Immunology, PathWest, Perth, Australia
A*24:638	DKMS‐LSL_ID20032311	OZ110998	DKMS Life Sciences Lab., Dresden, Germany
A*24:639	21QB010286	OZ004369	Dr Janette Kwok, Pokfulam Road, Hong Kong
A*24:640	21QB008957	OZ022572	Dr Janette Kwok, Pokfulam Road, Hong Kong
A*24:641N	DKMS‐LSL_ID20460411	OZ111008	DKMS Life Sciences Lab., Dresden, Germany
A*24:642	2024071621	PQ215530	Dr Mingrui Huo, Beijing, China
A*25:90	KSR151129	PP997981	Dr Maria Loginova, Kirov, Russia
A*25:91	KSR159264	PP997982	Dr Maria Loginova, Kirov, Russia
A*25:92	KSR182415	PQ168565	Dr Maria Loginova, Kirov, Russia
A*26:01:85	21QB005659	OZ022570	Dr Janette Kwok, Pokfulam Road, Hong Kong
A*26:250	21070930161	PQ113343	Dr Cathi Murphey, San Antonio, USA
A*26:251Q	24817892	PQ140476	Dr Luis A Marin, Albacete, Spain
A*26:252N	KSR203874	PQ168566	Dr Maria Loginova, Kirov, Russia
A*29:02:01:41	16‐10597	PP889337	Dr M Rosa Moya‐Quiles, El Palmar, Spain
A*29:196	21QB004715	OZ022569	Dr Janette Kwok, Pokfulam Road, Hong Kong
A*29:197	22042237641	PQ113344	Dr Cathi Murphey, San Antonio, USA
A*29:198	146559690	PQ119831	Dr Pascal Pedini, Marseille, France
A*30:01:30	21QB006913	OZ022571	Dr Janette Kwok, Pokfulam Road, Hong Kong
A*30:02:01:24	24‐0217	PP869413	Dr M Rosa Moya‐Quiles, El Palmar, Spain
A*30:231	DKMS‐LSL_ID20462796	OZ110992	DKMS Life Sciences Lab., Dresden, Germany
A*30:232	22QB010222	OY998279	Dr Janette Kwok, Pokfulam Road, Hong Kong
A*31:01:58	XRQ‐001	PP782800	Dr Cheng Yan Fan, Beijing, China
A*32:01:59	2410448180	PP928394	Dr Jonathan Visentin, Bordeaux, France
A*32:01:60	KSR177057	PP997984	Dr Maria Loginova, Kirov, Russia
A*32:01:61	22103143396	PQ145186	Dr Cathi Murphey, San Antonio, USA
A*32:190	61026156	PQ030910	Dr Dolores Planelles, Valencia, Spain
A*33:01:01:19	71613560	PP993589	Dr Luis A Marin, Albacete, Spain
A*33:03:69	20QB011771	OZ004358	Dr Janette Kwok, Pokfulam Road, Hong Kong
A*33:03:70	21QB008486	OZ004366	Dr Janette Kwok, Pokfulam Road, Hong Kong
A*33:70:02	240701_4304018	PQ066737	Dr Hyewon Park, Seoul, South Korea
A*33:242:01:02	19QB002763	OY998275	Dr Janette Kwok, Pokfulam Road, Hong Kong
A*33:273Q	DKMS‐LSL_ID20176725	OZ111002	DKMS Life Sciences Lab., Dresden, Germany
A*33:274	KSR139350	PQ168567	Dr Maria Loginova, Kirov, Russia
A*34:01:01:04	KWO010 (KAPA)	OZ121140	Anthony Nolan Research Institute, London, United Kingdom
A*36:15	6174353_LAS	PP943124	Dr Jeane Visentainer, Maringa, Brazil
A*66:49	KSR142426	PQ168568	Dr Maria Loginova, Kirov, Russia
A*68:01:02:36	DKMS‐LSL_ID20453565	OZ111007	DKMS Life Sciences Lab., Dresden, Germany
A*68:01:67	73210649762	PQ014357	Dr Pascal Pedini, Marseille, France
A*68:02:25	133495493	PQ119833	Dr Pascal Pedini, Marseille, France
A*74:01:11	LIB_6151755	PP996040	Ms Anaregina Araujo, Teresina, Brazil
A*74:51	2399396	PP910678	Dr Jose Samuel da Silva, Aparecida de Goiania Goias, Brazil
A*80:01:01:03	35020	OZ121123	Anthony Nolan Research Institute, London, United Kingdom
B*07:02:106	NTV_5966809	PP986963	Dr Georgia Daniela Marcusso, Porto Velho, Brazil
B*07:02:107	KSR158411	PQ168569	Dr Maria Loginova, Kirov, Russia
B*07:05:13	21QB000933	OZ004362	Dr Janette Kwok, Pokfulam Road, Hong Kong
B*07:10:02	HG00052203	PP904457	Histogenetics, Ossining, USA
B*07:516	KSR189272	PP997876	Dr Maria Loginova, Kirov, Russia
B*07:517	INCA1069617	PQ179479	Mr Vinicius Stelet, Rio de Janeiro, Brazil
B*08:01:01:75	AN303341	OZ076320	Anthony Nolan Research Institute, London, United Kingdom
B*08:01:78	KSR151474	PQ168570	Dr Maria Loginova, Kirov, Russia
B*08:325	NGR0106A, NGR0106B	PQ050370	Dr Laura Bungener, Groningen, The Netherlands
B*08:326	96117447	PQ067513	Dr Daniel Fuerst, Ulm, Germany
B*13:02:44	2022011140	OM965697	Dr Mingrui Huo, Beijing, China
B*13:200	21QB010515	OZ004370	Dr Janette Kwok, Pokfulam Road, Hong Kong
B*13:201	KSR140617	PQ168571	Dr Maria Loginova, Kirov, Russia
B*13:202	2022033155	PQ247470	Dr Mingrui Huo, Beijing, China
B*14:02:31:02	145879682	PQ119830	Dr Pascal Pedini, Marseille, France
B*15:01:88	RP‐285928	PP989837	Dr Neifi Hassan Saloum Deghaide, Ribeirao Preto, Brazil
B*15:01:89	KSR151270	PQ168572	Dr Maria Loginova, Kirov, Russia
B*15:04:05	NTV_6096261	PQ195598	Dr Georgia Daniela Marcusso, Porto Velho, Brazil
B*15:716	21QR006805	OY998284	Dr Janette Kwok, Pokfulam Road, Hong Kong
B*15:717	21QB007465	OZ004365	Dr Janette Kwok, Pokfulam Road, Hong Kong
B*15:718	21QR008940	OZ022574	Dr Janette Kwok, Pokfulam Road, Hong Kong
B*15:719	CMC‐E‐008	PQ066021	Mrs So‐Jung Choi, Seoul, South Korea
B*18:245N	HG00052211	PP947527	Histogenetics, Ossining, USA
B*18:246	73221283241	PQ014353	Dr Pascal Pedini, Marseille, France
B*18:247	137077912	PQ014350	Dr Pascal Pedini, Marseille, France
B*18:248N	KSR172878	PQ168573	Dr Maria Loginova, Kirov, Russia
B*18:249	22110843641	PQ145187	Dr Cathi Murphey, San Antonio, USA
B*18:250	NTV_6087172	PQ195597	Dr Georgia Daniela Marcusso, Porto Velho, Brazil
B*27:02:07	HG00051571	OR664312	Histogenetics, Ossining, USA
B*27:05:02:43	AN303323	OZ075068	Anthony Nolan Research Institute, London, United Kingdom
B*27:281	HG00052204	PP904458	Histogenetics, Ossining, USA
B*27:282	KSR197798	PQ168574	Dr Maria Loginova, Kirov, Russia
B*27:283	ID H144799	PQ212955	Miss Sriprapai Khanuntong, Bangkok, Thailand
B*35:01:01:89	AN303339	OZ076318	Anthony Nolan Research Institute, London, United Kingdom
B*35:03:01:38	24817913	PQ119494	Dr Luis A Marin, Albacete, Spain
B*35:03:43	2323205	PP855505	Dr Ina Skaljic, Chicago, USA
B*35:03:44	73210841156	PQ014356	Dr Pascal Pedini, Marseille, France
B*35:20:03	6000655‐EFS	PQ009210	Dr Jeane Visentainer, Maringa, Brazil
B*35:617	rdkm205110	PP501816	Ms Anastasiia Ananeva, Moscow, Russia
B*35:618	FSIMM_IMM‐20‐24072	OZ077738	Clinical Immunology, PathWest, Perth, Australia
B*35:619	148638945	PQ014356	Dr Pascal Pedini, Marseille, France
B*35:620	509996	PQ153117	Mr Alessandro Pirri, Curitiba, Brazil
B*35:621	NTV_6099340	PQ195600	Dr Georgia Daniela Marcusso, Porto Velho, Brazil
B*38:01:25	61022288	PP855884	Dr Dolores Planelles, Valencia, Spain
B*38:02:14	HG00052207	PP926145	Histogenetics, Ossining, USA
B*38:119	HG00052206	PP926144	Histogenetics, Ossining, USA
B*39:222	rdkm208102	PP501815	Ms Anastasiia Ananeva, Moscow, Russia
B*39:223	KSR117451	PP997877	Dr Maria Loginova, Kirov, Russia
B*39:224	20QB001606	OY998280	Dr Janette Kwok, Pokfulam Road, Hong Kong
B*39:225	021769681	PQ124027	Mrs Ornella Senoussi, La Tronche, France
B*39:226	KSR171002	PQ168575	Dr Maria Loginova, Kirov, Russia
B*40:01:90	kSR144965	PP997878	Dr Maria Loginova, Kirov, Russia
B*40:02:40	FSIMM_IMM‐24‐28036	OZ077757	Clinical Immunology, PathWest, Perth, Australia
B*40:06:34	20QB008496	OY998281	Dr Janette Kwok, Pokfulam Road, Hong Kong
B*40:585	H5724	PP874466	Mrs Na Liu, Beijing, China
B*40:586	HG00052210	PP947526	Histogenetics, Ossining, USA
B*40:587	2401847	PP986397	Dr Paul Rouzaire, Clermont‐Ferrand, France
B*40:588	19QB008295	OY998298	Dr Janette Kwok, Pokfulam Road, Hong Kong
B*40:589	21QB010301	OY998285	Dr Janette Kwok, Pokfulam Road, Hong Kong
B*40:590N	21QB001474	OZ004363	Dr Janette Kwok, Pokfulam Road, Hong Kong
B*40:591	21QB010033	OZ004367	Dr Janette Kwok, Pokfulam Road, Hong Kong
B*40:592	20QB013293	OZ004359	Dr Janette Kwok, Pokfulam Road, Hong Kong
B*40:593	KSR181186	PQ168576	Dr Maria Loginova, Kirov, Russia
B*40:594N	20240422067	PQ212879	Dr Mingrui Huo, Beijing, China
B*40:595	DG806494	PQ241116	Dr Cathi Murphey, San Antonio, USA
B*40:596	20240407088	PQ212878	Dr Mingrui Huo, Beijing, China
B*41:87	GR‐EVA‐58922	PP936195	Dr Diamanto Kouniaki, Athens, Greece
B*42:02:01:03	BST111420213	PP887623	Dr Francesc Rudilla, Barcelona, Spain
B*42:38	138398551	PQ014352	Dr Pascal Pedini, Marseille, France
B*44:02:01:86	AN303324	OZ075069	Anthony Nolan Research Institute, London, United Kingdom
B*44:02:01:87	AN303343	OZ076322	Anthony Nolan Research Institute, London, United Kingdom
B*44:03:01:75	DOP‐ND	OZ121133	Anthony Nolan Research Institute, London, United Kingdom
B*44:03:01:75	FH13	OZ121137	Anthony Nolan Research Institute, London, United Kingdom
B*44:03:72	HG00052205	PP904459	Histogenetics, Ossining, USA
B*44:409	HG00052212	PP947528	Histogenetics, Ossining, USA
B*44:410	901597112	PP868645	Mr Thomas Vaudois, Strasbourg, Frances
B*44:411	SMLab20240528_10889	WS100911	Dr Oh‐Joong Kwon, Seoul, South Korea
B*44:412	2024050349b	PP928994	Mr Benjamin Asghar, Chapel Hill, USA
B*44:413	20102213424‐RS	PQ043877	Dr Cathi Murphey, San Antonio, USA
B*45:36	23011845514	PQ182805	Dr Cathi Murphey, San Antonio, USA
B*46:01:43	2022021102	OM965698	Dr Mingrui Huo, Beijing, China
B*49:01:01:22	202402080401	PP418877	Dr Ram Mohan Jaiswal, Jaipur, India
B*49:01:27	901931837	PP852892	Mr Thomas Vaudois, Strasbourg, Frances
B*50:27N	KFU2764724	OR801765	Ms Anastasiia Ananeva, Moscow, Russia
B*51:01:01:123	AN303338	OZ076317	Anthony Nolan Research Institute, London, United Kingdom
B*51:01:111	NTV_5973295	PP996046	Dr Georgia Daniela Marcusso, Porto Velho, Brazil
B*51:237:03	19QB008081	OY998278	Dr Janette Kwok, Pokfulam Road, Hong Kong
B*51:415N	901477214	PP852891	Mr Thomas Vaudois, Strasbourg, Frances
B*51:416	SNUH‐E‐006	PQ243127	Mrs So‐Jung Choi, Seoul, South Korea
B*52:01:58	KSR113519	PQ168577	Dr Maria Loginova, Kirov, Russia
B*52:126	2462043	PP887423	Mrs Ana Lucia Rodrigues, Aparecida de Goiania, Brazil
B*52:127	2395264	PP910676	Dr Jose Samuel da Silva, Aparecida de Goiania Goias, Brazil
B*53:80N	KSR204513	PP997879	Dr Maria Loginova, Kirov, Russia
B*54:01:13	2023101754	PQ241458	Dr Mingrui Huo, Beijing, China
B*54:49N	KSR104235	PQ168578	Dr Maria Loginova, Kirov, Russia
B*54:50	2024010586	PQ197040	Dr Mingrui Huo, Beijing, China
B*55:141	P15325‐1	PQ118950	Prof Faming Zhu, Hangzhou, China
B*56:01:01:20	5344271	PP928414	Prof Renata Zunec, Zagreb, Croatia
B*57:01:01:31	AN303342	OZ076321	Anthony Nolan Research Institute, London, United Kingdom
B*57:185Q	HG00052202	PP904456	Histogenetics, Ossining, USA
B*58:01:49	20QB010132	OY998282	Dr Janette Kwok, Pokfulam Road, Hong Kong
B*58:156	81349610	PQ213472	Dr Daniel Fuerst, Ulm, Germany
B*81:14	HG00052208	PP926146	Histogenetics, Ossining, USA
B*81:15	78507199	PQ043879	Dr Cathi Murphey, San Antonio, USA
C*01:02:01:76	19QB006135	OY998306	Dr Janette Kwok, Pokfulam Road, Hong Kong
C*01:02:01:77	AN303329	OZ075415	Anthony Nolan Research Institute, London, United Kingdom
C*01:02:94	21QB002260	OY998299	Dr Janette Kwok, Pokfulam Road, Hong Kong
C*01:127:02	HG00052170	PP848421	Histogenetics, Ossining, USA
C*01:276	21QB004261	OY998300	Dr Janette Kwok, Pokfulam Road, Hong Kong
C*01:277	22QB005953	OY998302	Dr Janette Kwok, Pokfulam Road, Hong Kong
C*01:278	22QB008298	OY998304	Dr Janette Kwok, Pokfulam Road, Hong Kong
C*01:279N	22QB003882	OZ004372	Dr Janette Kwok, Pokfulam Road, Hong Kong
C*02:02:79	FSIMM_IMM‐24‐27951	OZ077751	Clinical Immunology, PathWest, Perth, Australia
C*02:247	1875092	PP853483	Mrs Elizabeth M. F. Leal Domingues, Aparecida de Goiania, Brazil
C*02:248	rdkm140155	PP914024	Ms Anastasiia Ananeva, Moscow, Russia
C*02:249	rdkm097159	PP914025	Ms Anastasiia Ananeva, Moscow, Russia
C*02:250	rdkm386167	PP914028	Ms Anastasiia Ananeva, Moscow, Russia
C*03:02:34	21QB007934	OY998301	Dr Janette Kwok, Pokfulam Road, Hong Kong
C*03:03:01:71	24011857126HS28053	PP887438	Dr Cathi Murphey, San Antonio, USA
C*03:03:71	7814329	PP355695	Ms Vibhuti Rambani, Bangalore, India
C*03:04:113	21QB000041	OZ004360	Dr Janette Kwok, Pokfulam Road, Hong Kong
C*03:04:114	rdkm420168	PP914030	Ms Anastasiia Ananeva, Moscow, Russia
C*03:09:02:02	573028	PP910669	Mrs Tamara Passos, Salvador, Brazil
C*03:309:02	1892224	PP910671	Dr Jose Samuel da Silva, Aparecida de Goiania Goias, Brazil
C*03:688	115895	PP763830	Dr Francisco de Paula Sanchez Gordo, Malaga, Spain
C*03:689	LYY‐001	PP782607	Dr Yu Jie Wen, Beijing, China
C*03:690	rdkm810147	PP501811	Ms Anastasiia Ananeva, Moscow, Russia
C*03:691	FSIMM_IMM‐24‐27983	OZ077753	Clinical Immunology, PathWest, Perth, Australia
C*03:692	2404854	PP910679	Dr Jose Samuel da Silva, Aparecida de Goiania Goias, Brazil
C*03:693	rdkm932152	PP914022	Ms Anastasiia Ananeva, Moscow, Russia
C*03:694	KSR124062	PQ034525	Dr Maria Loginova, Kirov, Russia
C*03:695	22QB000579	OZ004371	Dr Janette Kwok, Pokfulam Road, Hong Kong
C*03:696	RP‐283844	PQ181545	Dr Neifi Hassan Saloum Deghaide, Ribeirao Preto, Brazil
C*03:697N	KUMC‐E‐002	PQ243128	Mrs So‐Jung Choi, Seoul, South Korea
C*04:01:01:193	AN303326	OZ075071	Anthony Nolan Research Institute, London, United Kingdom
C*04:01:166	HG00052077	PP746990	Histogenetics, Ossining, USA
C*04:01:167	HG00052076	PP746989	Histogenetics, Ossining, USA
C*04:03:01:06	KWO010 (KAPA)	OZ121141	Anthony Nolan Research Institute, London, United Kingdom
C*04:32:01:02	24‐0112	PP869415	Dr M Rosa Moya‐Quiles, El Palmar, Spain
C*04:528	HG00052221	PP947516	Histogenetics, Ossining, USA
C*04:529	rdkm581152	PP914021	Ms Anastasiia Ananeva, Moscow, Russia
C*04:530	21QB000691	OZ004361	Dr Janette Kwok, Pokfulam Road, Hong Kong
C*04:531	6859	PP974322	Dr Daria Kilina, St Petersburg, Russia
C*04:532	rdkm569164	PP914027	Ms Anastasiia Ananeva, Moscow, Russia
C*05:01:01:93	AN303325	OZ075070	Anthony Nolan Research Institute, London, United Kingdom
C*05:299	HG00052222	PP947517	Histogenetics, Ossining, USA
C*05:300	HG00052171	PP848422	Histogenetics, Ossining, USA
C*05:301	KSR179734	PQ034526	Dr Maria Loginova, Kirov, Russia
C*05:302	21012125852	PQ182806	Dr Cathi Murphey, San Antonio, USA
C*06:02:117	rdkm723136	PP501813	Ms Anastasiia Ananeva, Moscow, Russia
C*06:02:118	rdkm711167	PP914029	Ms Anastasiia Ananeva, Moscow, Russia
C*06:44:02	BJ21342ZA, BJ21342ZABS	PP839795	Dr Zorana Andric, Belgrade, Serbia
C*06:388	HG00052219	PP926151	Histogenetics, Ossining, USA
C*06:389	FSIMM_IMM‐24‐27901	OZ077746	Clinical Immunology, PathWest, Perth, Australia
C*06:390	9000623544488092	PP976124	Prof Faming Zhu, Hangzhou, China
C*06:391	KFSH‐D_2401222694	PQ001010	Dr Rabab AlAttas, Dammam, Saudi Arabia
C*06:392	137082151	PQ014351	Dr Pascal Pedini, Marseille, France
C*06:393	137074620	PQ014348	Dr Pascal Pedini, Marseille, France
C*06:394	23051249172	PQ182807	Dr Cathi Murphey, San Antonio, USA
C*07:01:01:142	24‐1525	PP869419	Dr M Rosa Moya‐Quiles, El Palmar, Spain
C*07:01:01:143	71613515	PP921914	Dr Luis A Marin, Albacete, Spain
C*07:01:01:144	AN303330	OZ075416	Anthony Nolan Research Institute, London, United Kingdom
C*07:01:127	5344891	PP928406	Prof Renata Zunec, Zagreb, Croatia
C*07:01:128	137081804	PQ014346	Dr Pascal Pedini, Marseille, France
C*07:02:01:204	AN303331	OZ075417	Anthony Nolan Research Institute, London, United Kingdom
C*07:02:156	rdkm711136	PP501814	Ms Anastasiia Ananeva, Moscow, Russia
C*07:02:157	FSIMM_IMM‐24‐27928	OZ077748	Clinical Immunology, PathWest, Perth, Australia
C*07:02:158	rdkm331136	PP501827	Ms Anastasiia Ananeva, Moscow, Russia
C*07:02:159	rdkm084151	PP914020	Ms Anastasiia Ananeva, Moscow, Russia
C*07:02:160	20QB011326	OY998288	Dr Janette Kwok, Pokfulam Road, Hong Kong
C*07:18:13	73221375912	PQ014355	Dr Pascal Pedini, Marseille, France
C*07:1091:01:02	rdkm171173	PP947782	Ms Anastasiia Ananeva, Moscow, Russia
C*07:1152	317673	PP840851	Dr Raquel Fabreti, Belo Horizonte, Brazil
C*07:1153	rdkm584145	PP501812	Ms Anastasiia Ananeva, Moscow, Russia
C*07:1154	rdkm768142	PP898041	Ms Anastasiia Ananeva, Moscow, Russia
C*07:1155N	901289884	PP852893	Mr Thomas Vaudois, Strasbourg, Frances
C*07:1156	HG00052113	PP803078	Histogenetics, Ossining, USA
C*07:1157	rdkm859148	PP914018	Ms Anastasiia Ananeva, Moscow, Russia
C*07:1158	rdkm343152	PP914020	Ms Anastasiia Ananeva, Moscow, Russia
C*07:1159	LIB_6162807	PP996043	Ms Anaregina Araujo, Teresina, Brazil
C*07:1160Q	KSR178455	PQ034527	Dr Maria Loginova, Kirov, Russia
C*07:1161	137077891	PQ014349	Dr Pascal Pedini, Marseille, France
C*07:1162	BMD3486	PQ212619	Dr Cathi Murphey, San Antonio, USA
C*07:1163N	HS27147‐1	PQ243595	Dr Cathi Murphey, San Antonio, USA
C*08:01:41	21QB005414	OZ004364	Dr Janette Kwok, Pokfulam Road, Hong Kong
C*08:04:01:05	23‐2238	PP869416	Dr M Rosa Moya‐Quiles, El Palmar, Spain
C*08:291	23‐2485	PP841922	Dr M Rosa Moya‐Quiles, El Palmar, Spain
C*08:292Q	HG00052224	PP947519	Histogenetics, Ossining, USA
C*08:293	HG00052217	PP904464	Histogenetics, Ossining, USA
C*08:294	2024TB12411	PQ110139	Dr Keming Du, Shanghai, China
C*12:02:02:27	KWO010 (KAPA)	OZ121142	Anthony Nolan Research Institute, London, United Kingdom
C*12:03:01:68	23‐2663	PP869414	Dr M Rosa Moya‐Quiles, El Palmar, Spain
C*12:03:01:69	24‐0749	PP869418	Dr M Rosa Moya‐Quiles, El Palmar, Spain
C*12:03:01:70	0789107297S	PQ043871	Dr Cathi Murphey, San Antonio, USA
C*12:03:01:71	AN303019	OZ079880	Anthony Nolan Research Institute, London, United Kingdom
C*12:03:01:71	APA	OZ121129	Anthony Nolan Research Institute, London, United Kingdom
C*12:03:01:72	24817851	PQ119493	Dr Luis A Marin, Albacete, Spain
C*12:03:91	HG00052213	PP904460	Histogenetics, Ossining, USA
C*12:03:92	rdkm109155	PP914023	Ms Anastasiia Ananeva, Moscow, Russia
C*12:199:02	HG00052075	PP746988	Histogenetics, Ossining, USA
C*12:422	HG00052223	PP947518	Histogenetics, Ossining, USA
C*12:423	HG00052116	PP803081	Histogenetics, Ossining, USA
C*12:424	PP964009	PP964009	Mr Jonathon Glover, Philadelphia, USA
C*12:425	21QB010229	OZ004368	Dr Janette Kwok, Pokfulam Road, Hong Kong
C*12:426	rdkm752172	PP947781	Ms Anastasiia Ananeva, Moscow, Russia
C*12:427	81342965	PQ067514	Dr Daniel Fuerst, Ulm, Germany
C*14:02:01:36	24817862	PQ140478	Dr Luis A Marin, Albacete, Spain
C*15:02:01:66	GR‐EVA‐58436	PQ063872	Dr Diamanto Kouniaki, Athens, Greece
C*15:05:21	FSIMM_IMM‐24‐27985	OZ077754	Clinical Immunology, PathWest, Perth, Australia
C*15:05:22	rdkm711169	PP947780	Ms Anastasiia Ananeva, Moscow, Russia
C*15:152:04	HG00052216	PP904463	Histogenetics, Ossining, USA
C*15:283	HG00052214	PP904461	Histogenetics, Ossining, USA
C*15:284	HG00052115	PP803080	Histogenetics, Ossining, USA
C*15:285	HG00052169	PP826692	Histogenetics, Ossining, USA
C*15:286	19QB006135	OY998274	Dr Janette Kwok, Pokfulam Road, Hong Kong
C*15:287	20QB003039	OY998286	Dr Janette Kwok, Pokfulam Road, Hong Kong
C*15:288	20QB011205	OY998287	Dr Janette Kwok, Pokfulam Road, Hong Kong
C*15:289	2023011052	OR083234	Dr Mingrui Huo, Beijing, China
C*16:01:01:41	24‐0006	PP869417	Dr M Rosa Moya‐Quiles, El Palmar, Spain
C*17:80	138393541	PQ014347	Dr Pascal Pedini, Marseille, France
C*17:81	JH0133	PQ152311	Dr Maria Bettinotti, Baltimore, USA
E*01:01:01:63	2359713	PP555578	Dr Francesc Rudilla, Barcelona, Spain
E*01:01:01:64	AN303376	OZ079914	Anthony Nolan Research Institute, London, United Kingdom
E*01:01:44	STR2309167	PQ150390	Prof Faming Zhu, Hangzhou, China
E*01:03:01:16	AN303344	OZ076323	Anthony Nolan Research Institute, London, United Kingdom
E*01:03:02:41	AN303340	OZ076319	Anthony Nolan Research Institute, London, United Kingdom
E*01:151	STR2303775	PQ150389	Prof Faming Zhu, Hangzhou, China
F*01:01:01:45	24011857126HS28053	PP887439	Dr Cathi Murphey, San Antonio, USA
F*01:01:01:46	APA, KWO010 (KAPA)	OZ079942, OZ079967	Anthony Nolan Research Institute, London, United Kingdom
F*01:01:01:47	KWO010 (KAPA)	OZ079941	Anthony Nolan Research Institute, London, United Kingdom
F*01:01:01:48	PSCDA0504	PQ230459	Dr Seik‐Soon Khor, Tokyo, Japan
F*01:01:01:49	PSCDA1157	PQ230460	Dr Seik‐Soon Khor, Tokyo, Japan
F*01:01:01:50	PSCDA0078	PQ230461	Dr Seik‐Soon Khor, Tokyo, Japan
F*01:01:01:51	PSCDA0908	PQ230462	Dr Seik‐Soon Khor, Tokyo, Japan
F*01:01:01:52	PSCDA0836	PQ230464	Dr Seik‐Soon Khor, Tokyo, Japan
F*01:01:01:53	PSCDA0600	PQ261165	Dr Seik‐Soon Khor, Tokyo, Japan
F*01:01:01:54	PSCDA0366	PQ261166	Dr Seik‐Soon Khor, Tokyo, Japan
F*01:01:02:15	PSCDA1146	PQ261167	Dr Seik‐Soon Khor, Tokyo, Japan
F*01:05:01:02	PSCDA0326	PQ230463	Dr Seik‐Soon Khor, Tokyo, Japan
G*01:01:01:38	1521497	PP555586	Dr Francesc Rudilla, Barcelona, Spain
G*01:01:03:06	AN303337	OZ076324	Anthony Nolan Research Institute, London, United Kingdom
G*01:01:09:02	FH13	OZ079940	Anthony Nolan Research Institute, London, United Kingdom
G*01:01:32	2024040192	PP928995	Mr Benjamin Asghar, Chapel Hill, USA
G*01:01:33	KWO010 (KAPA), RSA‐ND	OZ079943, OZ079955	Anthony Nolan Research Institute, London, United Kingdom
G*01:24:02	HL‐58	PP533447	Dr Krishna Chaaithanya Itta, Mumbai, India
G*01:57	35020	OZ079876	Anthony Nolan Research Institute, London, United Kingdom
H*01:01:01:07	BGE	OZ079917	Anthony Nolan Research Institute, London, United Kingdom
H*01:01:01:08	BUR,E	OZ079918	Anthony Nolan Research Institute, London, United Kingdom
H*01:01:01:09	KY	OZ079944	Anthony Nolan Research Institute, London, United Kingdom
H*01:11	DM02753	PQ047483	Dr Cathi Murphey, San Antonio, USA
H*01:12	AWELLS	OZ079916	Anthony Nolan Research Institute, London, United Kingdom
H*01:13	35020	OZ079877	Anthony Nolan Research Institute, London, United Kingdom
H*02:01:01:05	AN303020	OZ079881	Anthony Nolan Research Institute, London, United Kingdom
H*02:03:02:02	AN301077	OZ079879	Anthony Nolan Research Institute, London, United Kingdom
H*02:07:02	AN303021	OZ079882	Anthony Nolan Research Institute, London, United Kingdom
H*02:08:01:03	DOP‐ND	OZ079939	Anthony Nolan Research Institute, London, United Kingdom
DRB1*01:154	HG00052260	PP926155	Histogenetics, Ossining, USA
DRB1*03:217	AN303320	OZ038309	Anthony Nolan Research Institute, London, United Kingdom
DRB1*03:218N	GR‐EVA‐59022	PQ037256	Dr Diamanto Kouniaki, Athens, Greece
DRB1*04:01:01:26	AN303336	OZ075423	Anthony Nolan Research Institute, London, United Kingdom
DRB1*04:04:01:15	AN303333	OZ075420	Anthony Nolan Research Institute, London, United Kingdom
DRB1*04:04:21	KSR125413	OR900519	Dr Maria Loginova, Kirov, Russia
DRB1*04:387	24022658422HS28209	PP887436	Dr Cathi Murphey, San Antonio, USA
DRB1*04:388	H5833	PP874467	Mrs Na Liu, Beijing, China
DRB1*04:389N	HG00052259	PP926154	Histogenetics, Ossining, USA
DRB1*07:163N	HS25533	PQ218340	Dr Cathi Murphey, San Antonio, USA
DRB1*08:02:07	HS29062	PQ241114	Dr Cathi Murphey, San Antonio, USA
DRB1*08:131	KSR109644	PP997986	Dr Maria Loginova, Kirov, Russia
DRB1*09:60	900062188101091	PQ118952	Prof Faming Zhu, Hangzhou, China
DRB1*11:01:49	9000624503229091	PQ118953	Prof Faming Zhu, Hangzhou, China
DRB1*11:01:50	DM2324HS24473	PQ218341	Dr Cathi Murphey, San Antonio, USA
DRB1*11:04:01:10	AN303335	OZ075422	Anthony Nolan Research Institute, London, United Kingdom
DRB1*11:101:04	LIB_6178046	PQ156401	Ms Anaregina Araujo, Teresina, Brazil
DRB1*11:335	24031459100HS24646	PP887441	Dr Cathi Murphey, San Antonio, USA
DRB1*11:336	HG00052256	PP904481	Histogenetics, Ossining, USA
DRB1*11:337	90458134022056	PP951068	Dr Antonio Balas, Madrid, Spain
DRB1*11:338	61028079	PQ030909	Dr Dolores Planelles, Valencia, Spain
DRB1*12:01:13	KSR109050	PP273455	Dr Maria Loginova, Kirov, Russia
DRB1*12:02:14	9000624502363093	PQ118954	Prof Faming Zhu, Hangzhou, China
DRB1*12:02:15	9000623544182092	PQ118955	Prof Faming Zhu, Hangzhou, China
DRB1*12:112	29000950	PP869275	Dr Daniel Fuerst, Ulm, Germany
DRB1*12:113	EUMC001	LC822848	Dr Soo‐Kyung Kim, Seoul, Republic of Korea
DRB1*13:363	967002995	PQ154481	Miss Sriprapai Khanuntong, Bangkok, Thailand
DRB1*14:268	2310620829	PQ213004	Dr Jonathan Visentin, Bordeaux, France
DRB1*14:269N	42053008	PQ246267	Dr Romain Ferru‐Clement, Poitiers, France
DRB1*15:01:01:32Q	10234	PP107972	Dr Marija Velickovic, Sydney, Australia
DRB1*15:01:52	G231222018	OZ060538	Miss Xingjie Li, Chengdu, China
DRB1*15:233	KSR190200	PP997987	Dr Maria Loginova, Kirov, Russia
DRB1*16:02:12	G220104007	OZ060531	Miss Xingjie Li, Chengdu, China
DRB1*16:02:13	AB8632	PP953612	Prof Edward KL Yang, Hualien, Taiwan
DRB1*16:77N	G20170824012	OZ060530	Miss Xingjie Li, Chengdu, China
DRB1*16:78	HG00052258	PP926153	Histogenetics, Ossining, USA
DRB1*16:79	UERJ‐1V220	PQ199712	Dr Romulo Vianna, Rio de Janeiro, Brazil
DRB3*01:130	HG00052262	PP947524	Histogenetics, Ossining, USA
DRB3*02:210Q	2024040192	PP938416	Mr Benjamin Asghar, Chapel Hill, USA
DRB3*02:211	d13613	PQ241113	Dr Cathi Murphey, San Antonio, USA
DRB3*02:212	C7194	PQ241115	Dr Cathi Murphey, San Antonio, USA
DRB3*03:73	2023033015	OR634968	Mr Jobson Nascimento, Recife, Brazil
DRB3*03:74	HG00052261	PP926156	Histogenetics, Ossining, USA
DRB3*03:75	021860483	PP951502	Mrs Ornella Senoussi, La Tronche, France
DRB3*03:76	JH0148	PQ227096	Dr Maria Bettinotti, Baltimore, USA
DRB4*01:03:01:19	PSCDA1028	PQ066359	Dr Seik‐Soon Khor, Tokyo, Japan
DRB4*01:03:01:20	PSCDA1146	PQ066360	Dr Seik‐Soon Khor, Tokyo, Japan
DRB4*01:186	FSIMM_IMM‐23‐81685	OZ077743	Clinical Immunology, PathWest, Perth, Australia
DRB4*01:187	RP‐285737	PP949238	Dr Neifi Hassan Saloum Deghaide, Ribeirao Preto, Brazil
DRB4*01:188	20QR006369	OY998290	Dr Janette Kwok, Pokfulam Road, Hong Kong
DRB5*01:142	61025308	PP968759	Dr Dolores Planelles, Valencia, Spain
DRB5*02:02:05	2402251	PP986399	Dr Paul Rouzaire, Clermont‐Ferrand, France
DQA1*01:02:28	021849943	PP796502	Mrs Ornella Senoussi, La Tronche, France
DQA1*01:04:10	42401050	PQ246268	Dr Romain Ferru‐Clement, Poitiers, France
DQA1*01:67:02	2410188529	PP854398	Dr Jonathan Visentin, Bordeaux, France
DQA1*01:161	HG00050835	OQ203998	Histogenetics, Ossining, USA
DQA1*01:162	HG00050843	OQ267727	Histogenetics, Ossining, USA
DQA1*01:163	FSIMM_IMM‐24‐27956	OZ077752	Clinical Immunology, PathWest, Perth, Australia
DQA1*01:164	RP‐281246	PP949240	Dr Neifi Hassan Saloum Deghaide, Ribeirao Preto, Brazil
DQA1*01:165	21QR001658	OY998291	Dr Janette Kwok, Pokfulam Road, Hong Kong
DQA1*01:166	23QR001794	OY998296	Dr Janette Kwok, Pokfulam Road, Hong Kong
DQA1*01:167	OT01611	PQ043875	Dr Cathi Murphey, San Antonio, USA
DQA1*01:168	PP951503	PP951503	Mrs Ornella Senoussi, La Tronche, France
DQA1*01:169	2410324697	PP952035	Dr Jonathan Visentin, Bordeaux, France
DQA1*01:170	2410148118	PP952036	Dr Jonathan Visentin, Bordeaux, France
DQA1*01:171	JH0135	PQ177844	Dr Maria Bettinotti, Baltimore, USA
DQA1*01:172	JH0136	PQ177845	Dr Maria Bettinotti, Baltimore, USA
DQA1*02:01:01:12	24819137	PQ156036	Dr Luis A Marin, Albacete, Spain
DQA1*02:46	0999124009840X	PP955039	Mr Kieran Robertson, Barnsley, United Kingdom
DQA1*02:47	78820724835	PQ043874	Dr Cathi Murphey, San Antonio, USA
DQA1*03:01:01:05	24817734	PQ009009	Dr Luis A Marin, Albacete, Spain
DQA1*03:01:01:06	24817546	PQ064105	Dr Luis A Marin, Albacete, Spain
DQA1*03:01:16	RP‐281908	PP974458	Dr Neifi Hassan Saloum Deghaide, Ribeirao Preto, Brazil
DQA1*03:01:17	61026227	PQ030911	Dr Dolores Planelles, Valencia, Spain
DQA1*03:02:06	9000624503100092	PQ118956	Prof Faming Zhu, Hangzhou, China
DQA1*03:78	11900001154	PP358852	Dr Francesc Rudilla, Barcelona, Spain
DQA1*03:79	222700300759	PP496482	Dr Ahmed Gaballa, Stockholm, Sweden
DQA1*03:80	GR‐EVA‐58132	PP737721	Dr Diamanto Kouniaki, Athens, Greece
DQA1*03:81	JH0138	PQ196099	Dr Maria Bettinotti, Baltimore, USA
DQA1*04:01:01:14	UERJ‐C20246	PQ066377	Dr Romulo Vianna, Rio de Janeiro, Brazil
DQA1*04:23	0789107297S	PQ043876	Dr Cathi Murphey, San Antonio, USA
DQA1*04:24	JH0141	PQ196102	Dr Maria Bettinotti, Baltimore, USA
DQA1*04:25	JH0142	PQ196103	Dr Maria Bettinotti, Baltimore, USA
DQA1*05:01:19	9000624503459092	PQ118957	Prof Faming Zhu, Hangzhou, China
DQA1*05:03:01:04	UERJ‐C20222	PQ066378	Dr Romulo Vianna, Rio de Janeiro, Brazil
DQA1*05:05:01:43	UERJ‐C20258	PQ066379	Dr Romulo Vianna, Rio de Janeiro, Brazil
DQA1*05:05:23	04797274	PP967216	Dr Antonio Balas, Madrid, Spain
DQA1*05:116Q	FSIMM_IMM‐24‐28029	OZ077756	Clinical Immunology, PathWest, Perth, Australia
DQA1*05:117	24212887	PP934379	Dr Daniel Fuerst, Ulm, Germany
DQA1*06:01:04	donor 622700301444	PQ035167	Dr Ahmed Gaballa, Stockholm, Sweden
DQA1*06:11	HG00052240	PP904477	Histogenetics, Ossining, USA
DQB1*05:01:01:38	DKMS‐LSL_ID21415814	OZ053379	DKMS Life Sciences Lab., Dresden, Germany
DQB1*05:01:01:39	DKMS‐LSL_ID21550201	OZ053364	DKMS Life Sciences Lab., Dresden, Germany
DQB1*05:01:51	DKMS‐LSL_ID21438033	OZ053373	DKMS Life Sciences Lab., Dresden, Germany
DQB1*05:01:52	DKMS‐LSL_ID20813727	OZ053335	DKMS Life Sciences Lab., Dresden, Germany
DQB1*05:01:53	DKMS‐LSL_ID21037359	OZ057515	DKMS Life Sciences Lab., Dresden, Germany
DQB1*05:01:54	DKMS‐LSL_ID21035702	OZ057516	DKMS Life Sciences Lab., Dresden, Germany
DQB1*05:02:01:19	GR‐EVA‐58985	PP936199	Dr Diamanto Kouniaki, Athens, Greece
DQB1*05:02:01:20	71613591	PP993593	Dr Luis A Marin, Albacete, Spain
DQB1*05:02:30	DKMS‐LSL_ID21479544	OZ053370	DKMS Life Sciences Lab., Dresden, Germany
DQB1*05:03:01:12	DKMS‐LSL_ID21089837	OZ053345	DKMS Life Sciences Lab., Dresden, Germany
DQB1*05:03:33	DKMS‐LSL_ID20807075	OZ053334	DKMS Life Sciences Lab., Dresden, Germany
DQB1*05:03:34	DKMS‐LSL_ID21256790	OZ057502	DKMS Life Sciences Lab., Dresden, Germany
DQB1*05:03:35	DKMS‐LSL_ID21574078, DKMS‐LSL_ID21074426	OZ057475, OZ057511	DKMS Life Sciences Lab., Dresden, Germany
DQB1*05:03:36	DKMS‐LSL_ID21646223	OZ057470	DKMS Life Sciences Lab., Dresden, Germany
DQB1*05:03:37	DKMS‐LSL_ID21647842, DKMS‐LSL_ID21185795	OZ057468, OZ057505	DKMS Life Sciences Lab., Dresden, Germany
DQB1*05:336	G230303001	OZ060542	Miss Xingjie Li, Chengdu, China
DQB1*05:337Q	DKMS‐LSL_ID21088317	OZ053344	DKMS Life Sciences Lab., Dresden, Germany
DQB1*05:338	DKMS‐LSL_ID21480949	OZ053369	DKMS Life Sciences Lab., Dresden, Germany
DQB1*05:339	DKMS‐LSL_ID21424508	OZ053376	DKMS Life Sciences Lab., Dresden, Germany
DQB1*05:340	DKMS‐LSL_ID21299380	OZ053393	DKMS Life Sciences Lab., Dresden, Germany
DQB1*05:341	DKMS‐LSL_ID21497677	OZ053367	DKMS Life Sciences Lab., Dresden, Germany
DQB1*05:342	DKMS‐LSL_ID21187365	OZ053356	DKMS Life Sciences Lab., Dresden, Germany
DQB1*05:343	DKMS‐LSL_ID21111765	OZ053349	DKMS Life Sciences Lab., Dresden, Germany
DQB1*05:344	DKMS‐LSL_ID21221198	OZ057504	DKMS Life Sciences Lab., Dresden, Germany
DQB1*05:345	DKMS‐LSL_ID20913772	OZ057460	DKMS Life Sciences Lab., Dresden, Germany
DQB1*05:346	DKMS‐LSL_ID20962005	OZ057521	DKMS Life Sciences Lab., Dresden, Germany
DQB1*05:347N	DKMS‐LSL_ID20600758	OZ057427	DKMS Life Sciences Lab., Dresden, Germany
DQB1*05:348N	DKMS‐LSL_ID20586366	OZ057446	DKMS Life Sciences Lab., Dresden, Germany
DQB1*05:349	DKMS‐LSL_ID21512630	OZ057485	DKMS Life Sciences Lab., Dresden, Germany
DQB1*05:350	HG00052251	PP926157	Histogenetics, Ossining, USA
DQB1*05:351	KFSH_2402061124	PQ001011	Dr Rabab AlAttas, Dammam, Saudi Arabia
DQB1*05:352	24216650	PQ114733	Dr Daniel Fuerst, Ulm, Germany
DQB1*06:01:01:09	GR‐EVA‐54707	PP936200	Dr Diamanto Kouniaki, Athens, Greece
DQB1*06:01:36	DKMS‐LSL_ID21285978	OZ053396	DKMS Life Sciences Lab., Dresden, Germany
DQB1*06:02:63	DKMS‐LSL_ID20804018	OZ053332	DKMS Life Sciences Lab., Dresden, Germany
DQB1*06:02:64	DKMS‐LSL_ID21014635	OZ053342	DKMS Life Sciences Lab., Dresden, Germany
DQB1*06:03:01:24	DKMS‐LSL_ID20931833	OZ053317	DKMS Life Sciences Lab., Dresden, Germany
DQB1*06:03:01:25	24817744	PQ009010	Dr Luis A Marin, Albacete, Spain
DQB1*06:03:01:26	UERJ‐C20185	PQ066381	Dr Romulo Vianna, Rio de Janeiro, Brazil
DQB1*06:03:51	rdkm261170	OR810608	Ms Anastasiia Ananeva, Moscow, Russia
DQB1*06:03:52	DKMS‐LSL_ID21485801	OZ053368	DKMS Life Sciences Lab., Dresden, Germany
DQB1*06:03:53	DKMS‐LSL_ID21570163	OZ057476	DKMS Life Sciences Lab., Dresden, Germany
DQB1*06:04:01:10	UERJ‐C20159	PQ066380	Dr Romulo Vianna, Rio de Janeiro, Brazil
DQB1*06:493	G221230008	OZ060541	Miss Xingjie Li, Chengdu, China
DQB1*06:494	017031	PP858889	Dr Lia Mele, Alessandria, Italy
DQB1*06:495	DKMS‐LSL_ID21101375	OZ053347	DKMS Life Sciences Lab., Dresden, Germany
DQB1*06:496	DKMS‐LSL_ID21351016	OZ053384	DKMS Life Sciences Lab., Dresden, Germany
DQB1*06:497	DKMS‐LSL_ID21350617	OZ053385	DKMS Life Sciences Lab., Dresden, Germany
DQB1*06:498	DKMS‐LSL_ID20975758	OZ053319	DKMS Life Sciences Lab., Dresden, Germany
DQB1*06:499	DKMS‐LSL_ID20978523	OZ053340	DKMS Life Sciences Lab., Dresden, Germany
DQB1*06:500	DKMS‐LSL_ID21435229	OZ053325	DKMS Life Sciences Lab., Dresden, Germany
DQB1*06:501	DKMS‐LSL_ID21521405, DKMS‐LSL_ID21304640	OZ053365, OZ057498	DKMS Life Sciences Lab., Dresden, Germany
DQB1*06:502	DKMS‐LSL_ID21108189, DKMS‐LSL_ID21325534	OZ053320, OZ053322	DKMS Life Sciences Lab., Dresden, Germany
DQB1*06:503	DKMS‐LSL_ID21172927	OZ053354	DKMS Life Sciences Lab., Dresden, Germany
DQB1*06:504	DKMS‐LSL_ID21222046	OZ053361	DKMS Life Sciences Lab., Dresden, Germany
DQB1*06:505	DKMS‐LSL_ID21468346	OZ057488	DKMS Life Sciences Lab., Dresden, Germany
DQB1*06:506	DKMS‐LSL_ID20924399, DKMS‐LSL_ID20934044	OZ057462, OZ057465	DKMS Life Sciences Lab., Dresden, Germany
DQB1*06:507N	DKMS‐LSL_ID21041402	OZ057433	DKMS Life Sciences Lab., Dresden, Germany
DQB1*06:508	DKMS‐LSL_ID21482870	OZ057436	DKMS Life Sciences Lab., Dresden, Germany
DQB1*06:509	DKMS‐LSL_ID21642760	OZ057471	DKMS Life Sciences Lab., Dresden, Germany
DQB1*06:510	DKMS‐LSL_ID20889679, DKMS‐LSL_ID21529275	OZ057458, OZ057482	DKMS Life Sciences Lab., Dresden, Germany
DQB1*06:511	DKMS‐LSL_ID20868630	OZ057453	DKMS Life Sciences Lab., Dresden, Germany
DQB1*06:512	DKMS‐LSL_ID21616966, DKMS‐LSL_ID20868551, DKMS‐LSL_ID21214492, DKMS‐LSL_ID21631916	OZ053338, OZ057435, OZ057441, OZ057443	DKMS Life Sciences Lab., Dresden, Germany
DQB1*06:513	DKMS‐LSL_ID20849891, DKMS‐LSL_ID21504806	OZ057451, OZ057486	DKMS Life Sciences Lab., Dresden, Germany
DQB1*06:514	DKMS‐LSL_ID20847135	OZ057450	DKMS Life Sciences Lab., Dresden, Germany
DQB1*06:515	DKMS‐LSL_ID21522530	OZ057438	DKMS Life Sciences Lab., Dresden, Germany
DQB1*06:516	DKMS‐LSL_ID21448307	OZ057490	DKMS Life Sciences Lab., Dresden, Germany
DQB1*06:517	DKMS‐LSL_ID21392907	OZ057494	DKMS Life Sciences Lab., Dresden, Germany
DQB1*06:518	93135468	PQ124122	Dr Daniel Fuerst, Ulm, Germany
DQB1*06:519	172310661	PQ119832	Dr Pascal Pedini, Marseille, France
DQB1*02:01:01:23	DKMS‐LSL_ID21357459	OZ053383	DKMS Life Sciences Lab., Dresden, Germany
DQB1*02:01:01:24	DKMS‐LSL_ID21190575	OZ057434	DKMS Life Sciences Lab., Dresden, Germany
DQB1*02:01:01:25	AN303327	OZ075424	Anthony Nolan Research Institute, London, United Kingdom
DQB1*02:01:48	DKMS‐LSL_ID21205280	OZ053359	DKMS Life Sciences Lab., Dresden, Germany
DQB1*02:01:49	DKMS‐LSL_ID21165358	OZ057507	DKMS Life Sciences Lab., Dresden, Germany
DQB1*02:01:50	FSIMM_IMM‐23‐76674	OZ077742	Clinical Immunology, PathWest, Perth, Australia
DQB1*02:02:01:23	AN303326	OZ075413	Anthony Nolan Research Institute, London, United Kingdom
DQB1*02:02:26	DKMS‐LSL_ID21369834	OZ053381	DKMS Life Sciences Lab., Dresden, Germany
DQB1*02:02:27	DKMS‐LSL_ID21647197	OZ057469	DKMS Life Sciences Lab., Dresden, Germany
DQB1*02:02:28	DKMS‐LSL_ID21012407	OZ057519	DKMS Life Sciences Lab., Dresden, Germany
DQB1*02:02:29	DKMS‐LSL_ID21512752	OZ057437	DKMS Life Sciences Lab., Dresden, Germany
DQB1*02:02:30	DKMS‐LSL_ID21550983	OZ057479	DKMS Life Sciences Lab., Dresden, Germany
DQB1*02:225Q	71605323	PP447455	Dr Cathi Murphey, San Antonio, USA
DQB1*02:226	DKMS‐LSL_ID21439048	OZ053372	DKMS Life Sciences Lab., Dresden, Germany
DQB1*02:227	DKMS‐LSL_ID20805725	OZ053333	DKMS Life Sciences Lab., Dresden, Germany
DQB1*02:228	DKMS‐LSL_ID20970574	OZ053339	DKMS Life Sciences Lab., Dresden, Germany
DQB1*02:229	DKMS‐LSL_ID21169631	OZ053353	DKMS Life Sciences Lab., Dresden, Germany
DQB1*02:230N	DKMS‐LSL_ID21213643	OZ053360	DKMS Life Sciences Lab., Dresden, Germany
DQB1*02:231	DKMS‐LSL_ID21076883	OZ057509	DKMS Life Sciences Lab., Dresden, Germany
DQB1*02:232	DKMS‐LSL_ID21257127	OZ057501	DKMS Life Sciences Lab., Dresden, Germany
DQB1*02:233	DKMS‐LSL_ID20920734	OZ057461	DKMS Life Sciences Lab., Dresden, Germany
DQB1*02:234	DKMS‐LSL_ID20925431	OZ057464	DKMS Life Sciences Lab., Dresden, Germany
DQB1*02:235	DKMS‐LSL_ID21554576	OZ057478	DKMS Life Sciences Lab., Dresden, Germany
DQB1*02:236	DKMS‐LSL_ID21077990	OZ057508	DKMS Life Sciences Lab., Dresden, Germany
DQB1*02:237	DKMS‐LSL_ID21374103	OZ057496	DKMS Life Sciences Lab., Dresden, Germany
DQB1*02:238	DKMS‐LSL_ID21384469	OZ057495	DKMS Life Sciences Lab., Dresden, Germany
DQB1*03:01:01:66	DKMS‐LSL_ID20766002	OZ053331	DKMS Life Sciences Lab., Dresden, Germany
DQB1*03:01:01:67	DKMS‐LSL_ID21488987	OZ057444	DKMS Life Sciences Lab., Dresden, Germany
DQB1*03:01:01:68	DKMS‐LSL_ID21543046	OZ057481	DKMS Life Sciences Lab., Dresden, Germany
DQB1*03:01:01:69	GR‐EVA‐58763	PP936203	Dr Diamanto Kouniaki, Athens, Greece
DQB1*03:01:01:70	24817855	PQ140479	Dr Luis A Marin, Albacete, Spain
DQB1*03:01:62	DKMS‐LSL_ID21463826	OZ053326	DKMS Life Sciences Lab., Dresden, Germany
DQB1*03:01:63	DKMS‐LSL_ID21336266	OZ053388	DKMS Life Sciences Lab., Dresden, Germany
DQB1*03:01:64	DKMS‐LSL_ID21248923	OZ057503	DKMS Life Sciences Lab., Dresden, Germany
DQB1*03:01:65	DKMS‐LSL_ID21436873	OZ057491	DKMS Life Sciences Lab., Dresden, Germany
DQB1*03:01:66	DKMS‐LSL_ID21015898	OZ057518	DKMS Life Sciences Lab., Dresden, Germany
DQB1*03:01:67	19QB004150	OY998338	Dr Janette Kwok, Pokfulam Road, Hong Kong
DQB1*03:01:68	021436665	PP621524	Dr Xavier Fournel, Saint Etienne, France
DQB1*03:02:01:26	DKMS‐LSL_ID21436873	OZ057492	DKMS Life Sciences Lab., Dresden, Germany
DQB1*03:02:01:27	FSIMM_IMM‐23‐49358	OZ077740	Clinical Immunology, PathWest, Perth, Australia
DQB1*03:02:01:28	24819166	PQ218690	Dr Luis A Marin, Albacete, Spain
DQB1*03:02:01:29	71616540	PQ241316	Dr Luis A Marin, Albacete, Spain
DQB1*03:02:42	DKMS‐LSL_ID21425308	OZ053375	DKMS Life Sciences Lab., Dresden, Germany
DQB1*03:02:43	DKMS‐LSL_ID21432320	OZ053374	DKMS Life Sciences Lab., Dresden, Germany
DQB1*03:02:44	DKMS‐LSL_ID20628097	OZ057447	DKMS Life Sciences Lab., Dresden, Germany
DQB1*03:02:45	DKMS‐LSL_ID21608567	OZ057474	DKMS Life Sciences Lab., Dresden, Germany
DQB1*03:02:46	DKMS‐LSL_ID20925277	OZ057463	DKMS Life Sciences Lab., Dresden, Germany
DQB1*03:03:02:17	DKMS‐LSL_ID20931833	OZ053318	DKMS Life Sciences Lab., Dresden, Germany
DQB1*03:03:02:18	DKMS‐LSL_ID20671686	OZ057448	DKMS Life Sciences Lab., Dresden, Germany
DQB1*03:03:02:19	71613531, 71613557	PP931990, PP993592	Dr Luis A Marin, Albacete, Spain
DQB1*03:03:02:20Q	19QR006724	OY998339	Dr Janette Kwok, Pokfulam Road, Hong Kong
DQB1*03:03:33	DKMS‐LSL_ID20732092	OZ057428	DKMS Life Sciences Lab., Dresden, Germany
DQB1*03:03:34	DKMS‐LSL_ID21625265	OZ057442	DKMS Life Sciences Lab., Dresden, Germany
DQB1*03:217:02	DKMS‐LSL_ID21516966	OZ057484	DKMS Life Sciences Lab., Dresden, Germany
DQB1*03:500:02	DKMS‐LSL_ID21487304	OZ057487	DKMS Life Sciences Lab., Dresden, Germany
DQB1*03:506:01:02	DKMS‐LSL_ID21338889	OZ053324	DKMS Life Sciences Lab., Dresden, Germany
DQB1*03:529	G220713016	OZ060535	Miss Xingjie Li, Chengdu, China
DQB1*03:530	G230921008	OZ060537	Miss Xingjie Li, Chengdu, China
DQB1*03:531	G211227008	OZ060539	Miss Xingjie Li, Chengdu, China
DQB1*03:532	G221108033	OZ060540	Miss Xingjie Li, Chengdu, China
DQB1*03:533	DKMS‐LSL_ID20749806	OZ053330	DKMS Life Sciences Lab., Dresden, Germany
DQB1*03:534	DKMS‐LSL_ID20561407	OZ053328	DKMS Life Sciences Lab., Dresden, Germany
DQB1*03:535	DKMS‐LSL_ID21025924, DKMS‐LSL_ID21267624	OZ053343, OZ057500	DKMS Life Sciences Lab., Dresden, Germany
DQB1*03:536	DKMS‐LSL_ID20996335	OZ053341	DKMS Life Sciences Lab., Dresden, Germany
DQB1*03:537	DKMS‐LSL_ID21303169	OZ053392	DKMS Life Sciences Lab., Dresden, Germany
DQB1*03:538	DKMS‐LSL_ID21292709	OZ053394	DKMS Life Sciences Lab., Dresden, Germany
DQB1*03:539	DKMS‐LSL_ID21336039	OZ053389	DKMS Life Sciences Lab., Dresden, Germany
DQB1*03:540	DKMS‐LSL_ID21285723	OZ053321	DKMS Life Sciences Lab., Dresden, Germany
DQB1*03:541N	DKMS‐LSL_ID21102184	OZ053348	DKMS Life Sciences Lab., Dresden, Germany
DQB1*03:542N	DKMS‐LSL_ID21337325	OZ053323	DKMS Life Sciences Lab., Dresden, Germany
DQB1*03:543	DKMS‐LSL_ID21198384	OZ053357	DKMS Life Sciences Lab., Dresden, Germany
DQB1*03:544	DKMS‐LSL_ID21054966	OZ057512	DKMS Life Sciences Lab., Dresden, Germany
DQB1*03:545	DKMS‐LSL_ID21306217	OZ057497	DKMS Life Sciences Lab., Dresden, Germany
DQB1*03:546	DKMS‐LSL_ID21047690	OZ057513	DKMS Life Sciences Lab., Dresden, Germany
DQB1*03:547	DKMS‐LSL_ID21276395	OZ057499	DKMS Life Sciences Lab., Dresden, Germany
DQB1*03:548	DKMS‐LSL_ID21619505	OZ057473	DKMS Life Sciences Lab., Dresden, Germany
DQB1*03:549	DKMS‐LSL_ID20897107	OZ057431	DKMS Life Sciences Lab., Dresden, Germany
DQB1*03:550	DKMS‐LSL_ID20974536	OZ057520	DKMS Life Sciences Lab., Dresden, Germany
DQB1*03:551N	DKMS‐LSL_ID20732763	OZ057429	DKMS Life Sciences Lab., Dresden, Germany
DQB1*03:552	DKMS‐LSL_ID20885062	OZ057457	DKMS Life Sciences Lab., Dresden, Germany
DQB1*03:553	DKMS‐LSL_ID21620130	OZ057472	DKMS Life Sciences Lab., Dresden, Germany
DQB1*03:554	DKMS‐LSL_ID20935517	OZ057466	DKMS Life Sciences Lab., Dresden, Germany
DQB1*03:555	DKMS‐LSL_ID20875506	OZ057454	DKMS Life Sciences Lab., Dresden, Germany
DQB1*03:556	DKMS‐LSL_ID20879002	OZ057455	DKMS Life Sciences Lab., Dresden, Germany
DQB1*03:557	DKMS‐LSL_ID20937276	OZ057467	DKMS Life Sciences Lab., Dresden, Germany
DQB1*03:558	DKMS‐LSL_ID20831372	OZ057449	DKMS Life Sciences Lab., Dresden, Germany
DQB1*03:559	DKMS‐LSL_ID21016082	OZ057517	DKMS Life Sciences Lab., Dresden, Germany
DQB1*03:560N	DKMS‐LSL_ID21518152	OZ057483	DKMS Life Sciences Lab., Dresden, Germany
DQB1*03:561	DKMS‐LSL_ID21405776	OZ057493	DKMS Life Sciences Lab., Dresden, Germany
DQB1*03:562	04797274	PP967215	Dr Antonio Balas, Madrid, Spain
DQB1*03:563	19QB003977	OY998305	Dr Janette Kwok, Pokfulam Road, Hong Kong
DQB1*03:564Q	22QB008123	OZ000553	Dr Janette Kwok, Pokfulam Road, Hong Kong
DQB1*03:565	61029712	PQ065631	Dr Dolores Planelles, Valencia, Spain
DQB1*04:01:08	22QB007778	OY998340	Dr Janette Kwok, Pokfulam Road, Hong Kong
DQB1*04:02:01:22	DKMS‐LSL_ID20879002	OZ057456	DKMS Life Sciences Lab., Dresden, Germany
DQB1*04:101	G231024019	OZ060543	Miss Xingjie Li, Chengdu, China
DQB1*04:102	DKMS‐LSL_ID20852059	OZ057452	DKMS Life Sciences Lab., Dresden, Germany
DQB1*04:103	DKMS‐LSL_ID21544822	OZ057480	DKMS Life Sciences Lab., Dresden, Germany
DQB1*04:104	UERJ‐C16398‐DQB1	PQ199713	Dr Romulo Vianna, Rio de Janeiro, Brazil
DPA1*01:03:01:92	incabrp0405	PP992532	Mr Vinicius Stelet, Rio de Janeiro, Brazil
DPA1*01:03:01:93	24817746	PQ009011	Dr Luis A Marin, Albacete, Spain
DPA1*01:03:01:94	71616460	PQ156037	Dr Luis A Marin, Albacete, Spain
DPA1*01:03:01:95	201_01119	PP961281	Ms Yuanli Zhao, Xi'an, China
DPA1*01:03:59	24053061827BMD78047082	PP887442	Dr Cathi Murphey, San Antonio, USA
DPA1*01:03:60	22QH000067	OY998303	Dr Janette Kwok, Pokfulam Road, Hong Kong
DPA1*01:04:01:02	24817763	PP911308	Dr Luis A Marin, Albacete, Spain
DPA1*01:209	24211022	PP869276	Dr Daniel Fuerst, Ulm, Germany
DPA1*01:210	HG00052229	PP926165	Histogenetics, Ossining, USA
DPA1*01:211	HG00052230	PP926166	Histogenetics, Ossining, USA
DPA1*01:212	HG00052082	PP747005	Histogenetics, Ossining, USA
DPA1*01:213	GR‐EVA‐58252	PP936196	Dr Diamanto Kouniaki, Athens, Greece
DPA1*01:214	GR‐EVA‐55565	PP737718	Dr Diamanto Kouniaki, Athens, Greece
DPA1*01:214	6009798‐VRT	PP943123	Dr Jeane Visentainer, Maringa, Brazil
DPA1*01:215	RP‐281241	PP949239	Dr Neifi Hassan Saloum Deghaide, Ribeirao Preto, Brazil
DPA1*01:216	RP‐281422	PP949241	Dr Neifi Hassan Saloum Deghaide, Ribeirao Preto, Brazil
DPA1*01:217N	NGR0095	PQ050368	Dr Laura Bungener, Groningen, The Netherlands
DPA1*01:218	73231304704	PQ119829	Dr Pascal Pedini, Marseille, France
DPA1*01:219N	JH0145	PQ202987	Dr Maria Bettinotti, Baltimore, USA
DPA1*01:220	71616514	PQ218689	Dr Luis A Marin, Albacete, Spain
DPA1*01:221	24817900	PQ241076	Dr Luis A Marin, Albacete, Spain
DPA1*02:01:01:36	GR‐EVA‐53600	PP737719	Dr Diamanto Kouniaki, Athens, Greece
DPA1*02:01:01:37	GR‐EVA‐58464	PP737720	Dr Diamanto Kouniaki, Athens, Greece
DPA1*02:01:01:38	71613557	PP993591	Dr Luis A Marin, Albacete, Spain
DPA1*02:01:01:39	201‐00502	PP961274	Ms Yuanli Zhao, Xi'an, China
DPA1*02:02:02:18	204‐01345	PP961278	Ms Yuanli Zhao, Xi'an, China
DPA1*02:02:02:19	204‐01061	PP961279	Ms Yuanli Zhao, Xi'an, China
DPA1*02:02:02:20	972	PP961280	Ms Yuanli Zhao, Xi'an, China
DPA1*02:02:16	22QR000267	OY998292	Dr Janette Kwok, Pokfulam Road, Hong Kong
DPA1*02:02:17	201‐00347	PP961271	Ms Yuanli Zhao, Xi'an, China
DPA1*02:02:18	JH0147	PQ227095	Dr Maria Bettinotti, Baltimore, USA
DPA1*02:03:06	814/23	PP682044	Ms Maria Ines Azevedo de Abreu, Vitoria, Brazil
DPA1*02:03:07	OT00548	PQ241117	Dr Cathi Murphey, San Antonio, USA
DPA1*02:131:01:02	22QR004396	OY998295	Dr Janette Kwok, Pokfulam Road, Hong Kong
DPA1*02:139	24‐1664	PP986905	Dr M Rosa Moya‐Quiles, El Palmar, Spain
DPA1*02:140N	24‐0647	PP986906	Dr M Rosa Moya‐Quiles, El Palmar, Spain
DPA1*02:141	inca1075514	PP992531	Mr Vinicius Stelet, Rio de Janeiro, Brazil
DPA1*02:142	incabrp0405	PP992533	Mr Vinicius Stelet, Rio de Janeiro, Brazil
DPA1*02:143	22QR004190	OY998294	Dr Janette Kwok, Pokfulam Road, Hong Kong
DPA1*02:144	23QR001796	OY998297	Dr Janette Kwok, Pokfulam Road, Hong Kong
DPA1*02:145	HD855	PQ043870	Dr Cathi Murphey, San Antonio, USA
DPA1*02:146	24N01084	NA114786	Mr Bin Chen, Tianjin, China
DPA1*03:01:01:16	UERJ‐C20190	PQ066375	Dr Romulo Vianna, Rio de Janeiro, Brazil
DPB1*01:01:14	DKMS‐LSL_ID21540267	OZ123305	DKMS Life Sciences Lab., Dresden, Germany
DPB1*01:01:15	PQ066763	PQ066763	Dr Prabhakar Putheti, Valhalla, USA
DPB1*01:01:16	DPB2419896A	PQ215784	Dr Ina Skaljic, Chicago, USA
DPB1*02:01:02:105	DKMS‐LSL_ID20708744	OZ125934	DKMS Life Sciences Lab., Dresden, Germany
DPB1*02:01:02:106	DKMS‐LSL_ID20653280	OZ125932	DKMS Life Sciences Lab., Dresden, Germany
DPB1*02:01:02:107	DKMS‐LSL_ID21430471	OZ123321	DKMS Life Sciences Lab., Dresden, Germany
DPB1*02:01:02:108	DKMS‐LSL_ID21464963	OZ123314	DKMS Life Sciences Lab., Dresden, Germany
DPB1*02:01:70	DKMS‐LSL_ID21182917	OZ125980	DKMS Life Sciences Lab., Dresden, Germany
DPB1*03:01:01:33	DKMS‐LSL_ID21365472	OZ125964	DKMS Life Sciences Lab., Dresden, Germany
DPB1*03:01:31	DKMS‐LSL_ID20974248	OZ125949	DKMS Life Sciences Lab., Dresden, Germany
DPB1*03:01:32	DKMS‐LSL_ID21050107	OZ123282	DKMS Life Sciences Lab., Dresden, Germany
DPB1*03:01:33	021896135	PQ124028	Mrs Ornella Senoussi, La Tronche, France
DPB1*04:01:01:153	AN303333	OZ075419	Anthony Nolan Research Institute, London, United Kingdom
DPB1*04:01:01:154	AN303334	OZ075421	Anthony Nolan Research Institute, London, United Kingdom
DPB1*04:01:01:155	DKMS‐LSL_ID21304421	OZ125972	DKMS Life Sciences Lab., Dresden, Germany
DPB1*04:01:01:156	DKMS‐LSL_ID20708744	OZ125935	DKMS Life Sciences Lab., Dresden, Germany
DPB1*04:01:01:157	DKMS‐LSL_ID20721942	OZ125938	DKMS Life Sciences Lab., Dresden, Germany
DPB1*04:01:01:158	DKMS‐LSL_ID20440670	OZ125913	DKMS Life Sciences Lab., Dresden, Germany
DPB1*04:01:01:159	DKMS‐LSL_ID20776909	OZ125941	DKMS Life Sciences Lab., Dresden, Germany
DPB1*04:01:01:160	DKMS‐LSL_ID20653280	OZ125933	DKMS Life Sciences Lab., Dresden, Germany
DPB1*04:01:01:161	DKMS‐LSL_ID21435140	OZ125957	DKMS Life Sciences Lab., Dresden, Germany
DPB1*04:01:01:162	DKMS‐LSL_ID20782053	OZ125942	DKMS Life Sciences Lab., Dresden, Germany
DPB1*04:01:78	DKMS‐LSL_ID21365472	OZ125965	DKMS Life Sciences Lab., Dresden, Germany
DPB1*04:01:79	DKMS‐LSL_ID20651535	OZ125931	DKMS Life Sciences Lab., Dresden, Germany
DPB1*04:01:80	DKMS‐LSL_ID20975772	OZ125950	DKMS Life Sciences Lab., Dresden, Germany
DPB1*04:01:81	DKMS‐LSL_ID21185168	OZ125979	DKMS Life Sciences Lab., Dresden, Germany
DPB1*04:01:82	DKMS‐LSL_ID20641309	OZ125926	DKMS Life Sciences Lab., Dresden, Germany
DPB1*04:01:83	DKMS‐LSL_ID20440670	OZ125912	DKMS Life Sciences Lab., Dresden, Germany
DPB1*04:01:84	DKMS‐LSL_ID21334477	OZ125969	DKMS Life Sciences Lab., Dresden, Germany
DPB1*04:01:85	DKMS‐LSL_ID20540079	OZ125915	DKMS Life Sciences Lab., Dresden, Germany
DPB1*04:01:86	DKMS‐LSL_ID20837153	OZ123264	DKMS Life Sciences Lab., Dresden, Germany
DPB1*04:01:87	DKMS‐LSL_ID20732549	OZ123258	DKMS Life Sciences Lab., Dresden, Germany
DPB1*04:01:88	DKMS‐LSL_ID21041292	OZ123281	DKMS Life Sciences Lab., Dresden, Germany
DPB1*04:01:89	DKMS‐LSL_ID21512257	OZ123308	DKMS Life Sciences Lab., Dresden, Germany
DPB1*04:01:90	DKMS‐LSL_ID21436333	OZ123320	DKMS Life Sciences Lab., Dresden, Germany
DPB1*04:01:91	DKMS‐LSL_ID21148351	OZ123332	DKMS Life Sciences Lab., Dresden, Germany
DPB1*04:01:92	DKMS‐LSL_ID21400108	OZ123323	DKMS Life Sciences Lab., Dresden, Germany
DPB1*04:02:01:45	AN303318	OZ038307	Anthony Nolan Research Institute, London, United Kingdom
DPB1*04:02:27	2410360171	PP928393	Dr Jonathan Visentin, Bordeaux, France
DPB1*04:02:28	DKMS‐LSL_ID21550573	OZ123302	DKMS Life Sciences Lab., Dresden, Germany
DPB1*04:02:29	DKMS‐LSL_ID20966145	OZ123280	DKMS Life Sciences Lab., Dresden, Germany
DPB1*04:02:30	DKMS‐LSL_ID21502927	OZ123310	DKMS Life Sciences Lab., Dresden, Germany
DPB1*04:02:31	DKMS‐LSL_ID21464963, DKMS‐LSL_ID21464960	OZ123315, OZ123316	DKMS Life Sciences Lab., Dresden, Germany
DPB1*04:02:32	DKMS‐LSL_ID21309557, DKMS‐LSL_ID21625916	OZ123296, OZ123327	DKMS Life Sciences Lab., Dresden, Germany
DPB1*05:01:01:40	DKMS‐LSL_ID21619201	OZ123298	DKMS Life Sciences Lab., Dresden, Germany
DPB1*05:01:24	DKMS‐LSL_ID21491320	OZ123312	DKMS Life Sciences Lab., Dresden, Germany
DPB1*06:01:01:09	AN303328	OZ075414	Anthony Nolan Research Institute, London, United Kingdom
DPB1*06:01:07	DKMS‐LSL_ID21511669	OZ123309	DKMS Life Sciences Lab., Dresden, Germany
DPB1*10:01:07	DKMS‐LSL_ID21111737	OZ123290	DKMS Life Sciences Lab., Dresden, Germany
DPB1*11:01:01:04	AN303332	OZ075418	Anthony Nolan Research Institute, London, United Kingdom
DPB1*11:01:08	DKMS‐LSL_ID21395953	OZ125962	DKMS Life Sciences Lab., Dresden, Germany
DPB1*13:01:01:18	DKMS‐LSL_ID20708751	OZ125936	DKMS Life Sciences Lab., Dresden, Germany
DPB1*14:01:16	DKMS‐LSL_ID20773347	OZ123261	DKMS Life Sciences Lab., Dresden, Germany
DPB1*15:01:08	DKMS‐LSL_ID21345871	OZ125966	DKMS Life Sciences Lab., Dresden, Germany
DPB1*19:01:01:08	5343674	PP928413	Prof Renata Zunec, Zagreb, Croatia
DPB1*26:01:02:04	DKMS‐LSL_ID21464960	OZ123317	DKMS Life Sciences Lab., Dresden, Germany
DPB1*104:01:01:17	5342597	PP928412	Prof Renata Zunec, Zagreb, Croatia
DPB1*104:01:09	DKMS‐LSL_ID20959986	OZ125946	DKMS Life Sciences Lab., Dresden, Germany
DPB1*105:01:01:20	DKMS‐LSL_ID21111197	OZ123288	DKMS Life Sciences Lab., Dresden, Germany
DPB1*425:01:02	DKMS‐LSL_ID20753474	OZ123259	DKMS Life Sciences Lab., Dresden, Germany
DPB1*650:01:01:02	AN303316	OZ038304	Anthony Nolan Research Institute, London, United Kingdom
DPB1*1290:01:01:02	DKMS‐LSL_ID21537266	OZ123306	DKMS Life Sciences Lab., Dresden, Germany
DPB1*1377:01:01:02	DKMS‐LSL_ID20892141	OZ123271	DKMS Life Sciences Lab., Dresden, Germany
DPB1*1639:01	539836	PP885724	Mrs Tamara Passos, Salvador, Brazil
DPB1*1640:01	901579751, 901580244	PP852896, PP852897	Mr Thomas Vaudois, Strasbourg, Frances
DPB1*1641:01	JN‐96, JN‐679	PP951996	Ass. Prof Cheng Bian, Kunming, China
DPB1*1642:01	HG00052235	PP926162	Histogenetics, Ossining, USA
DPB1*1643:01	HG00052233	PP904471	Histogenetics, Ossining, USA
DPB1*1644:01	HG00052231, HG00052232	PP904469, PP904470	Histogenetics, Ossining, USA
DPB1*1645:01N	HG00052234	PP926161	Histogenetics, Ossining, USA
DPB1*1646:01	HG00052238	PP947525	Histogenetics, Ossining, USA
DPB1*1647:01	HG00052237	PP926164	Histogenetics, Ossining, USA
DPB1*1648:01	FSIMM_IMM‐23‐95923	OZ077744	Clinical Immunology, PathWest, Perth, Australia
DPB1*1649:01	FSIMM_IMM‐23‐48837	OZ077739	Clinical Immunology, PathWest, Perth, Australia
DPB1*1650:01N	EUMC002	LC830767	Dr Soo‐Kyung Kim, Seoul, Republic of Korea
DPB1*1651:01	SZ‐232	PQ035126	Dr Hong‐Yan Zou, Shenzhen, China
DPB1*1652:01	22QR003504	OZ005802	Dr Janette Kwok, Pokfulam Road, Hong Kong
DPB1*1653:01	NGR0082	PP093764	Dr Laura Bungener, Groningen, The Netherlands
DPB1*1654:01	NGR0099	PP197225	Dr Laura Bungener, Groningen, The Netherlands
DPB1*1655:01	H240298‐1	PQ118958	Prof Faming Zhu, Hangzhou, China
DPB1*1656:01	9000624503760091	PQ118959	Prof Faming Zhu, Hangzhou, China
DPB1*1657:01	DKMS‐LSL_ID21335558	OZ125968	DKMS Life Sciences Lab., Dresden, Germany
DPB1*1658:01	DKMS‐LSL_ID21288139	OZ125973	DKMS Life Sciences Lab., Dresden, Germany
DPB1*1659:01	DKMS‐LSL_ID21223454	OZ125977	DKMS Life Sciences Lab., Dresden, Germany
DPB1*1660:01N	DKMS‐LSL_ID20622296	OZ125922	DKMS Life Sciences Lab., Dresden, Germany
DPB1*1661:01N	DKMS‐LSL_ID20631445	OZ125924	DKMS Life Sciences Lab., Dresden, Germany
DPB1*1662:01N	DKMS‐LSL_ID21284839	OZ125975	DKMS Life Sciences Lab., Dresden, Germany
DPB1*1663:01N	DKMS‐LSL_ID21418686	OZ125961	DKMS Life Sciences Lab., Dresden, Germany
DPB1*1664:01	DKMS‐LSL_ID21498338	OZ125954	DKMS Life Sciences Lab., Dresden, Germany
DPB1*1665:01	DKMS‐LSL_ID21432965	OZ125958	DKMS Life Sciences Lab., Dresden, Germany
DPB1*1666:01	DKMS‐LSL_ID20817230	OZ125944	DKMS Life Sciences Lab., Dresden, Germany
DPB1*1667:01N	DKMS‐LSL_ID20588072	OZ125918	DKMS Life Sciences Lab., Dresden, Germany
DPB1*1668:01	DKMS‐LSL_ID21467672	OZ125955	DKMS Life Sciences Lab., Dresden, Germany
DPB1*1669:01	DKMS‐LSL_ID21458907, DKMS‐LSL_ID20893151	OZ123272, OZ125956	DKMS Life Sciences Lab., Dresden, Germany
DPB1*1670:01	DKMS‐LSL_ID20709416, DKMS‐LSL_ID21367321	OZ123257, OZ125963	DKMS Life Sciences Lab., Dresden, Germany
DPB1*1671:01	DKMS‐LSL_ID21148784	OZ125982	DKMS Life Sciences Lab., Dresden, Germany
DPB1*1672:01	DKMS‐LSL_ID21175155	OZ125981	DKMS Life Sciences Lab., Dresden, Germany
DPB1*1673:01	DKMS‐LSL_ID20592230	OZ125919	DKMS Life Sciences Lab., Dresden, Germany
DPB1*1674:01	DKMS‐LSL_ID20601870	OZ125920	DKMS Life Sciences Lab., Dresden, Germany
DPB1*1675:01	DKMS‐LSL_ID21422039	OZ125960	DKMS Life Sciences Lab., Dresden, Germany
DPB1*1676:01	DKMS‐LSL_ID20637166	OZ125925	DKMS Life Sciences Lab., Dresden, Germany
DPB1*1677:01N	DKMS‐LSL_ID20621281	OZ125921	DKMS Life Sciences Lab., Dresden, Germany
DPB1*1678:01	DKMS‐LSL_ID21209367	OZ125978	DKMS Life Sciences Lab., Dresden, Germany
DPB1*1679:01	DKMS‐LSL_ID21271746	OZ125976	DKMS Life Sciences Lab., Dresden, Germany
DPB1*1680:01	DKMS‐LSL_ID20713225	OZ125937	DKMS Life Sciences Lab., Dresden, Germany
DPB1*1681:01	DKMS‐LSL_ID20631285	OZ125923	DKMS Life Sciences Lab., Dresden, Germany
DPB1*1682:01	DKMS‐LSL_ID20937156	OZ125945	DKMS Life Sciences Lab., Dresden, Germany
DPB1*1683:01	DKMS‐LSL_ID21017062	OZ125953	DKMS Life Sciences Lab., Dresden, Germany
DPB1*1684:01	DKMS‐LSL_ID20964832	OZ125947	DKMS Life Sciences Lab., Dresden, Germany
DPB1*1685:01	DKMS‐LSL_ID20497748	OZ125914	DKMS Life Sciences Lab., Dresden, Germany
DPB1*1686:01N	DKMS‐LSL_ID20972647	OZ125948	DKMS Life Sciences Lab., Dresden, Germany
DPB1*1687:01	DKMS‐LSL_ID20649510	OZ125929	DKMS Life Sciences Lab., Dresden, Germany
DPB1*1688:01	DKMS‐LSL_ID20787460, DKMS‐LSL_ID21286404	OZ125943, OZ125974	DKMS Life Sciences Lab., Dresden, Germany
DPB1*1689:01	DKMS‐LSL_ID20559854	OZ125916	DKMS Life Sciences Lab., Dresden, Germany
DPB1*1690:01	DKMS‐LSL_ID21327064	OZ125970	DKMS Life Sciences Lab., Dresden, Germany
DPB1*1691:01	DKMS‐LSL_ID21201124	OZ123331	DKMS Life Sciences Lab., Dresden, Germany
DPB1*1692:01	DKMS‐LSL_ID21237423	OZ123330	DKMS Life Sciences Lab., Dresden, Germany
DPB1*1693:01	DKMS‐LSL_ID21546418	OZ123303	DKMS Life Sciences Lab., Dresden, Germany
DPB1*1694:01	DKMS‐LSL_ID21619201	OZ123297	DKMS Life Sciences Lab., Dresden, Germany
DPB1*1695:01	DKMS‐LSL_ID20821890	OZ123262	DKMS Life Sciences Lab., Dresden, Germany
DPB1*1696:01	DKMS‐LSL_ID20687799	OZ123256	DKMS Life Sciences Lab., Dresden, Germany
DPB1*1697:01	DKMS‐LSL_ID21595339	OZ123299	DKMS Life Sciences Lab., Dresden, Germany
DPB1*1698:01	DKMS‐LSL_ID21544101	OZ123304	DKMS Life Sciences Lab., Dresden, Germany
DPB1*1699:01	DKMS‐LSL_ID21667214	OZ123291	DKMS Life Sciences Lab., Dresden, Germany
DPB1*1700:01	DKMS‐LSL_ID20825623	OZ123263	DKMS Life Sciences Lab., Dresden, Germany
DPB1*1701:01	DKMS‐LSL_ID20851537, DKMS‐LSL_ID21552097	OZ123267, OZ123301	DKMS Life Sciences Lab., Dresden, Germany
DPB1*1702:01	DKMS‐LSL_ID21629419	OZ123294	DKMS Life Sciences Lab., Dresden, Germany
DPB1*1703:01	DKMS‐LSL_ID21628146	OZ123295	DKMS Life Sciences Lab., Dresden, Germany
DPB1*1704:01	DKMS‐LSL_ID20844281	OZ123266	DKMS Life Sciences Lab., Dresden, Germany
DPB1*1705:01	DKMS‐LSL_ID20856387	OZ123268	DKMS Life Sciences Lab., Dresden, Germany
DPB1*1706:01	DKMS‐LSL_ID21529598	OZ123307	DKMS Life Sciences Lab., Dresden, Germany
DPB1*1707:01	DKMS‐LSL_ID21652285	OZ123292	DKMS Life Sciences Lab., Dresden, Germany
DPB1*1708:01	DKMS‐LSL_ID20856935	OZ123269	DKMS Life Sciences Lab., Dresden, Germany
DPB1*1709:01	DKMS‐LSL_ID21083717	OZ123284	DKMS Life Sciences Lab., Dresden, Germany
DPB1*1710:01	DKMS‐LSL_ID21495296	OZ123311	DKMS Life Sciences Lab., Dresden, Germany
DPB1*1711:01	DKMS‐LSL_ID21402248	OZ123322	DKMS Life Sciences Lab., Dresden, Germany
DPB1*1712:01	DKMS‐LSL_ID21088560	OZ123285	DKMS Life Sciences Lab., Dresden, Germany
DPB1*1713:01N	DKMS‐LSL_ID20948680	OZ123279	DKMS Life Sciences Lab., Dresden, Germany
DPB1*1714:01	DKMS‐LSL_ID20932359	OZ123277	DKMS Life Sciences Lab., Dresden, Germany
DPB1*1715:01	DKMS‐LSL_ID21384582	OZ123325	DKMS Life Sciences Lab., Dresden, Germany
DPB1*1716:01	DKMS‐LSL_ID21396905	OZ123324	DKMS Life Sciences Lab., Dresden, Germany
DPB1*1717:01	DKMS‐LSL_ID21453322	OZ123318	DKMS Life Sciences Lab., Dresden, Germany
DPB1*1718:01	DKMS‐LSL_ID20865162	OZ123270	DKMS Life Sciences Lab., Dresden, Germany
DPB1*1719:01	DKMS‐LSL_ID20920754	OZ123276	DKMS Life Sciences Lab., Dresden, Germany
DPB1*1720:01	DKMS‐LSL_ID21258150	OZ123329	DKMS Life Sciences Lab., Dresden, Germany
DPB1*1721:01	DKMS‐LSL_ID20894035, DKMS‐LSL_ID20914456	OZ123273, OZ123275	DKMS Life Sciences Lab., Dresden, Germany
DPB1*1722:01	DKMS‐LSL_ID21111197, DKMS‐LSL_ID21111198	OZ123287, OZ123289	DKMS Life Sciences Lab., Dresden, Germany
DPB1*1723:01	DKMS‐LSL_ID21585614	OZ123300	DKMS Life Sciences Lab., Dresden, Germany
DPB1*1724:01	DKMS‐LSL_ID21482690	OZ123313	DKMS Life Sciences Lab., Dresden, Germany
DPB1*1725:01	DKMS‐LSL_ID21315425	OZ123326	DKMS Life Sciences Lab., Dresden, Germany
DPB1*1726:01	DKMS‐LSL_ID20908459	OZ123274	DKMS Life Sciences Lab., Dresden, Germany
DPB1*1727:01	LIB_6174342	PQ156400	Ms Anaregina Araujo, Teresina, Brazil
DPB1*1728:01N	60990921	ON952475	Dr Dolores Planelles, Valencia, Spain
DPB1*1729:01:01:01	DKMS‐LSL_ID20978092	OZ125951	DKMS Life Sciences Lab., Dresden, Germany
DPB1*1729:01:01:02	DKMS‐LSL_ID20841070	OZ123265	DKMS Life Sciences Lab., Dresden, Germany
DMA*01:01:01:42	AN303357, AN303356	OZ079893, OZ079894	Anthony Nolan Research Institute, London, United Kingdom
DMB*01:01:01:50	AN303280, AN303363	OZ079883, OZ079900	Anthony Nolan Research Institute, London, United Kingdom
DMB*01:01:01:51	AN303348, AN303351, AN303362, AN303354, AN303361, AN300504, AN303359, AN303360, AN303365, AN303349	OZ079878, OZ079885, OZ079886, OZ079888, OZ079891, OZ079896, OZ079897, OZ079898, OZ079899, OZ079902	Anthony Nolan Research Institute, London, United Kingdom
DMB*01:03:01:19	AN303358, AN303375, AN303346, AN303374	OZ079895, OZ079912, OZ079913, OZ079966	Anthony Nolan Research Institute, London, United Kingdom
DOB*01:01:03:10	AN303367, AN303364	OZ079901, OZ079904	Anthony Nolan Research Institute, London, United Kingdom
DOB*01:04:02:02	AN303371, AN303368, AN303373, AN303352, AN303367, AN303370	OZ079889, OZ079905, OZ079906, OZ079908, OZ079909, OZ079911	Anthony Nolan Research Institute, London, United Kingdom
DOB*01:04:03:02	AN303350, AN303353, AN303366, AN303372, AN303369	OZ079887, OZ079890, OZ079903, OZ079907, OZ079910	Anthony Nolan Research Institute, London, United Kingdom
DOB*01:18N	AN303377	OZ079915	Anthony Nolan Research Institute, London, United Kingdom

**TABLE 2:** Confirmatory sequences.

HLA allele	Cell identification	Accession number	Submitting author
A*01:01:01:01	COX	OZ079919	Anthony Nolan Research Institute, London, United Kingdom
A*01:01:142	DKMS‐LSL_ID20037359	OZ110999	DKMS Life Sciences Lab., Dresden, Germany
A*01:01:161	JH0131	PQ152313	Dr Maria Bettinotti, Baltimore, USA
A*01:394N	DKMS‐LSL_ID20208841	OZ111003	DKMS Life Sciences Lab., Dresden, Germany
A*01:450N	DKMS‐LSL_ID21417201	OZ111011	DKMS Life Sciences Lab., Dresden, Germany
A*02:01:01:01	DBB	OZ079929	Anthony Nolan Research Institute, London, United Kingdom
A*02:01:01:173	DKMS‐LSL_ID20013782	OZ110996	DKMS Life Sciences Lab., Dresden, Germany
A*02:01:36	JMDP01K0415	LC822394	Dr Masahiro Satake, Tokyo, Japan
A*02:06:06	JMDP01K0402	LC822391	Dr Masahiro Satake, Tokyo, Japan
A*02:41	JMDP01K0408	LC822392	Dr Masahiro Satake, Tokyo, Japan
A*02:71	JMDP36K0529	LC822401	Dr Masahiro Satake, Tokyo, Japan
A*02:80	NT00529	PQ064531	Dr Jennifer Ng, Rockville, USA
A*02:107	KSR92770	PQ034507	Dr Maria Loginova, Kirov, Russia
A*02:110	FSIMM_IMM‐24‐27948	OZ077750	Clinical Immunology, PathWest, Perth, Australia
A*02:112	JMDP36K0522	LC822400	Dr Masahiro Satake, Tokyo, Japan
A*02:121	AA5‐6001640	NA114698	Mr Bin Chen, Tianjin, China
A*02:121	KSR129692	PQ034510	Dr Maria Loginova, Kirov, Russia
A*02:154	KSR113620	PQ034509	Dr Maria Loginova, Kirov, Russia
A*02:246	KSR144626	PQ034511	Dr Maria Loginova, Kirov, Russia
A*02:300	JMDP36K0491	LC822397	Dr Masahiro Satake, Tokyo, Japan
A*02:438	JMDP01K0331	LC822389	Dr Masahiro Satake, Tokyo, Japan
A*02:481	LIB_6182283	PP996039	Ms Anaregina Araujo, Teresina, Brazil
A*02:607	KSR113496	PQ034508	Dr Maria Loginova, Kirov, Russia
A*02:723	JMDP01K0252	LC822385	Dr Masahiro Satake, Tokyo, Japan
A*02:801	JMDP36K0497	LC822399	Dr Masahiro Satake, Tokyo, Japan
A*02:980	DKMS‐LSL_ID20264949	OZ111005	DKMS Life Sciences Lab., Dresden, Germany
A*02:1029	DKMS‐LSL_ID20582201	OZ111015	DKMS Life Sciences Lab., Dresden, Germany
A*02:1034	GR‐EVA‐58922	PP936198	Dr Diamanto Kouniaki, Athens, Greece
A*02:1133	20240506090	PQ212880	Dr Mingrui Huo, Beijing, China
A*02:1169	JH0130	PQ152312	Dr Maria Bettinotti, Baltimore, USA
A*02:1178	2024060378	PQ215529	Dr Mingrui Huo, Beijing, China
A*03:01:01:01	PGF	OZ079945	Anthony Nolan Research Institute, London, United Kingdom
A*03:01:56	NGR0105	PQ050369	Dr Laura Bungener, Groningen, The Netherlands
A*03:01:109	DKMS‐LSL_ID20664412	OZ111014	DKMS Life Sciences Lab., Dresden, Germany
A*03:38	NT00737	PQ015333	Dr Jennifer Ng, Rockville, USA
A*03:97	KSR177085	PQ034513	Dr Maria Loginova, Kirov, Russia
A*03:102	122404814	PQ066728	Dr Maria Peixoto, Porto, Portugal
A*03:187	2472339	PQ243968	Mrs Elizabeth M. F. Leal Domingues, Aparecida de Goiania, Brazil
A*03:456	DKMS‐LSL_ID20016936	OZ110997	DKMS Life Sciences Lab., Dresden, Germany
A*11:01:01:07	204‐00926	PP986995	Miss Yue Han, Xi'an, China
A*11:01:01:75	DKMS‐LSL_ID20536147	OZ111017	DKMS Life Sciences Lab., Dresden, Germany
A*11:15:01	JMDP36K0383	LC822387	Dr Masahiro Satake, Tokyo, Japan
A*11:22	NT00528	PQ015330	Dr Jennifer Ng, Rockville, USA
A*11:23	NT00516	PQ015329	Dr Jennifer Ng, Rockville, USA
A*11:33:01	JMDP36K0531	LC822402	Dr Masahiro Satake, Tokyo, Japan
A*11:112	KSR172770	PQ034512	Dr Maria Loginova, Kirov, Russia
A*23:139	DKMS‐LSL_ID20534581	OZ111018	DKMS Life Sciences Lab., Dresden, Germany
A*24:62	JMDP01K0264	LC822386	Dr Masahiro Satake, Tokyo, Japan
A*24:63	JMDP01K0367	LC822390	Dr Masahiro Satake, Tokyo, Japan
A*24:459:02	LIB_6093209	PQ240517	Ms Anaregina Araujo, Teresina, Brazil
A*24:534	DKMS‐LSL_ID19861692	OZ110993	DKMS Life Sciences Lab., Dresden, Germany
A*26:86	JMDP01K0492	LC822403	Dr Masahiro Satake, Tokyo, Japan
A*30:01:01:01	35020	OZ121122	Anthony Nolan Research Institute, London, United Kingdom
A*30:01:22	21QB002720	OY998277	Dr Janette Kwok, Pokfulam Road, Hong Kong
A*30:197N	DKMS‐LSL_ID20010588	OZ110995	DKMS Life Sciences Lab., Dresden, Germany
A*31:01:02:32	DKMS‐LSL_ID20549852	OZ111016	DKMS Life Sciences Lab., Dresden, Germany
A*31:01:25	JMDP01K0435	LC822398	Dr Masahiro Satake, Tokyo, Japan
A*31:29	JMDP36K0486	LC822396	Dr Masahiro Satake, Tokyo, Japan
A*31:161	JMDP36K0478	LC822395	Dr Masahiro Satake, Tokyo, Japan
A*32:01:01:01	SSTO	OZ079956	Anthony Nolan Research Institute, London, United Kingdom
A*32:14	NGR0107A, NGR0107B	PQ050371	Dr Laura Bungener, Groningen, The Netherlands
A*32:26:01	022112529	PQ241121	Mrs Ornella Senoussi, La Tronche, France
A*33:10	NT00715	PQ015332	Dr Jennifer Ng, Rockville, USA
A*33:10	JMDP36K0464	LC822393	Dr Masahiro Satake, Tokyo, Japan
A*34:02:01:01	FH13	OZ121135	Anthony Nolan Research Institute, London, United Kingdom
A*66:05	JMDP01K0315	LC822388	Dr Masahiro Satake, Tokyo, Japan
A*68:02:01:01	FH13	OZ121177	Anthony Nolan Research Institute, London, United Kingdom
A*68:28	NT00515	PQ015328	Dr Jennifer Ng, Rockville, USA
A*68:42	NT00752	PQ015331	Dr Jennifer Ng, Rockville, USA
A*68:264	DKMS‐LSL_ID20745994	OZ111012	DKMS Life Sciences Lab., Dresden, Germany
A*68:322	021850003	PP897948	Mrs Ornella Senoussi, La Tronche, France
A*74:26	JMDP36K0366	LC822237	Dr Masahiro Satake, Tokyo, Japan
B*07:02:01:01	PGF	OZ079946	Anthony Nolan Research Institute, London, United Kingdom
B*07:44N	116845	PP943410	Dr Francisco de Paula Sanchez Gordo, Malaga, Spain
B*08:01:01:01	COX	OZ079920	Anthony Nolan Research Institute, London, United Kingdom
B*08:01:14	KSR117144	PQ034514	Dr Maria Loginova, Kirov, Russia
B*08:33	BARB9689	PP994498	Dr Rafael Formenton Cita, Barretos, Brazil
B*13:01:01:12	BMT_3080_SARR	PP419904	Dr Samir Agarwal, New Delhi, India
B*13:150	KSR118932	PQ034515	Dr Maria Loginova, Kirov, Russia
B*13:198N	HG00052209	PP926147	Histogenetics, Ossining, USA
B*15:02:01:01	APA	OZ121174	Anthony Nolan Research Institute, London, United Kingdom
B*15:10:01:01	FH13	OZ121136	Anthony Nolan Research Institute, London, United Kingdom
B*15:16:01:02	DOP‐ND	OZ121175	Anthony Nolan Research Institute, London, United Kingdom
B*15:17:01:04	24817748	PQ046017	Dr Luis A Marin, Albacete, Spain
B*15:46	JMDP36K0467	LC830858	Dr Masahiro Satake, Tokyo, Japan
B*15:50	JMDP36K0524	LC830859	Dr Masahiro Satake, Tokyo, Japan
B*15:81	JMDP01K0425	LC830861	Dr Masahiro Satake, Tokyo, Japan
B*15:120	JMDP36K0534	LC830734	Dr Masahiro Satake, Tokyo, Japan
B*15:224	JMDP36K0469	LC830735	Dr Masahiro Satake, Tokyo, Japan
B*15:225	JMDP36K0451	LC830857	Dr Masahiro Satake, Tokyo, Japan
B*15:513	MDP36K0233	LC830860	Dr Masahiro Satake, Tokyo, Japan
B*18:145	BARB9853	PP994500	Dr Rafael Formenton Cita, Barretos, Brazil
B*35:01:01:05	35020	OZ121124	Anthony Nolan Research Institute, London, United Kingdom
B*35:01:01:55	5344964	PP928408	Prof Renata Zunec, Zagreb, Croatia
B*35:01:01:85	20QB010132	OY998283	Dr Janette Kwok, Pokfulam Road, Hong Kong
B*35:06	BARB9834	PP994499	Mrs Maristela Araujo Franca Mendes, Barretos, Brazil
B*35:35	JMDP36K0512	LC831078	Dr Masahiro Satake, Tokyo, Japan
B*35:60	JMDP01K0448	LC831079	Dr Masahiro Satake, Tokyo, Japan
B*35:92	JMDP01K0490	LC831080	Dr Masahiro Satake, Tokyo, Japan
B*35:131:02	JMDP36K0303	LC830862	Dr Masahiro Satake, Tokyo, Japan
B*35:609N	901179400	PP852890	Mr Thomas Vaudois, Strasbourg, Frances
B*39:01:01:28	AN302948	OZ026186	Anthony Nolan Research Institute, London, United Kingdom
B*39:107	JMDP01K0393	LC831081	Dr Masahiro Satake, Tokyo, Japan
B*39:188	HG00050583	OP661241	Histogenetics, Ossining, USA
B*39:219	NTV_5932096	PP975013	Dr Georgia Daniela Marcusso, Porto Velho, Brazil
B*40:01:02:11	201‐00009	PP932467	Miss Yue Han, Xi'an, China
B*40:13	24817857	PQ140477	Dr Luis A Marin, Albacete, Spain
B*40:33	JMDP01K0431	LC831085	Dr Masahiro Satake, Tokyo, Japan
B*40:53	JMDP01K0461	LC831087	Dr Masahiro Satake, Tokyo, Japan
B*40:67	JMDP01K0463	LC831088	Dr Masahiro Satake, Tokyo, Japan
B*40:78	473452	PP701657	Prof Edward KL Yang, Hualien, Taiwan
B*40:105	120424134	PQ112523	Prof Renata Zunec, Zagreb, Croatia
B*40:148	JMDP36K0474	LC831082	Dr Masahiro Satake, Tokyo, Japan
B*40:289	71613574	PP993595	Dr Luis A Marin, Albacete, Spain
B*40:307	JMDP36K0544	LC831083	Dr Masahiro Satake, Tokyo, Japan
B*40:325	JMDP01K0255	LC831084	Dr Masahiro Satake, Tokyo, Japan
B*40:465	JMDP01K0310	LC831086	Dr Masahiro Satake, Tokyo, Japan
B*40:592	22121944733	PQ145188	Dr Cathi Murphey, San Antonio, USA
B*41:02:01:05	24819001	PQ064107	Dr Luis A Marin, Albacete, Spain
B*44:02:01:01	SSTO	OZ079957	Anthony Nolan Research Institute, London, United Kingdom
B*44:39	021888787	PP951505	Mrs Ornella Senoussi, La Tronche, France
B*44:299	JMDP36K0515	LC831089	Dr Masahiro Satake, Tokyo, Japan
B*46:01:04	JMDP36K0499	LC831090	Dr Masahiro Satake, Tokyo, Japan
B*46:01:30	JMDP01K0307	LC831091	Dr Masahiro Satake, Tokyo, Japan
B*46:35	JMDP01K0418	LC831222	Dr Masahiro Satake, Tokyo, Japan
B*48:12	T4194	PP860611	Dr Carlos Vilches, Madrid, Spain
B*49:01:01:20Q	JH0132	PQ152310	Dr Maria Bettinotti, Baltimore, USA
B*51:01:04	71613555	PP993594	Dr Luis A Marin, Albacete, Spain
B*51:01:19	KSR172650	PQ034516	Dr Maria Loginova, Kirov, Russia
B*51:01:44	D302308	PP977174	Prof Edward KL Yang, Hualien, Taiwan
B*51:21	JMDP36K0282	LC831223	Dr Masahiro Satake, Tokyo, Japan
B*51:324	JMDP01K0348	LC831224	Dr Masahiro Satake, Tokyo, Japan
B*52:01:01:02	BGE	OZ121130	Anthony Nolan Research Institute, London, United Kingdom
B*52:01:17	JMDP36K0488	LC831225	Dr Masahiro Satake, Tokyo, Japan
B*52:12	JMDP01K0272	LC831226	Dr Masahiro Satake, Tokyo, Japan
B*52:14:02	JMDP01K0207	LC831227	Dr Masahiro Satake, Tokyo, Japan
B*52:39	JMDP36K0242	LC831228	Dr Masahiro Satake, Tokyo, Japan
B*54:25	JMDP01K0278	LC831229	Dr Masahiro Satake, Tokyo, Japan
B*54:42	JMDP01K0271	LC831230	Dr Masahiro Satake, Tokyo, Japan
B*55:02:01:04	APA	OZ121128	Anthony Nolan Research Institute, London, United Kingdom
B*55:07	JMDP01K0464	LC831231	Dr Masahiro Satake, Tokyo, Japan
B*55:22	AN16341097	PP486325	Anthony Nolan Research Institute, London, United Kingdom
B*55:32	JMDP01K0404	LC831232	Dr Masahiro Satake, Tokyo, Japan
B*55:39	470030	PP444721	Prof Edward KL Yang, Hualien, Taiwan
B*56:01:01:04	BUR,E	OZ121131	Anthony Nolan Research Institute, London, United Kingdom
B*57:01:01:01	DBB	OZ079930	Anthony Nolan Research Institute, London, United Kingdom
B*57:03:01:05	35020	OZ121125	Anthony Nolan Research Institute, London, United Kingdom
B*58:19	JMDP36K0272	LC831233	Dr Masahiro Satake, Tokyo, Japan
B*59:06	JMDP36K0516	LC831234	Dr Masahiro Satake, Tokyo, Japan
B*59:11	JMDP01K0226	LC831235	Dr Masahiro Satake, Tokyo, Japan
B*59:12	JMDP36K0270	LC831236	Dr Masahiro Satake, Tokyo, Japan
C*01:02:01:01	BUR,E	OZ121132	Anthony Nolan Research Institute, London, United Kingdom
C*01:02:01:74Q	8185344	PP329840	Ms Vibhuti Rambani, Bangalore, India
C*01:02:21	JMDP01K0355	LC831373	Dr Masahiro Satake, Tokyo, Japan
C*01:02:55	HG00019243	MK143726	Histogenetics, Ossining, USA
C*01:12:02	JMDP01K0412	LC831521	Dr Masahiro Satake, Tokyo, Japan
C*01:54	JMDP01K0495	LC831522	Dr Masahiro Satake, Tokyo, Japan
C*01:107	JMDP36K0426	LC831374	Dr Masahiro Satake, Tokyo, Japan
C*01:262	FG8856	PQ066766	Dr Thibault Pajot, Lille, France
C*01:272	71613501	PP921915	Dr Luis A Marin, Albacete, Spain
C*02:190	116793	PP943409	Dr Francisco de Paula Sanchez Gordo, Malaga, Spain
C*02:230	HG00052218	PP926150	Histogenetics, Ossining, USA
C*03:03:69	HG00052220	PP926152	Histogenetics, Ossining, USA
C*03:04:02:01	FH13	OZ121178	Anthony Nolan Research Institute, London, United Kingdom
C*03:04:03	71613549	PP993590	Dr Luis A Marin, Albacete, Spain
C*03:09:02:01	589698	PQ241451	Mrs Tamara Passos, Salvador, Brazil
C*03:54	KSR153713	PQ034517	Dr Maria Loginova, Kirov, Russia
C*03:78	JMDP36K0493	LC831523	Dr Masahiro Satake, Tokyo, Japan
C*03:97	JMDP01K0466	LC831524	Dr Masahiro Satake, Tokyo, Japan
C*03:301	LIB_5887605	PP996045	Ms Anaregina Araujo, Teresina, Brazil
C*03:626	HG00050590, HG00050591	OP661246, OP661247	Histogenetics, Ossining, USA
C*03:645	901810959	PP868644	Mr Thomas Vaudois, Strasbourg, Frances
C*04:01:01:14	DOP‐ND	OZ121134	Anthony Nolan Research Institute, London, United Kingdom
C*04:01:01:14	FH13	OZ121138	Anthony Nolan Research Institute, London, United Kingdom
C*04:01:01:75	35020	OZ121126	Anthony Nolan Research Institute, London, United Kingdom
C*04:01:30	JMDP36K0535	LC831525	Dr Masahiro Satake, Tokyo, Japan
C*04:124	022023607	PP951504	Mrs Ornella Senoussi, La Tronche, France
C*05:01:01:02	SSTO	OZ079958	Anthony Nolan Research Institute, London, United Kingdom
C*05:37	BARB9842	PP994501	Mrs Maristela Araujo Franca Mendes, Barretos, Brazil
C*06:02:01:01	DBB	OZ079931	Anthony Nolan Research Institute, London, United Kingdom
C*07:01:01:01	COX	OZ079921	Anthony Nolan Research Institute, London, United Kingdom
C*07:01:126	FG8632	OR604570	Dr Vincent Elsermans, Lille cedex, France
C*07:02:01:03	PGF	OZ079947	Anthony Nolan Research Institute, London, United Kingdom
C*07:02:50	HS27147‐1	PQ241112	Dr Cathi Murphey, San Antonio, USA
C*07:04:31	HG00052215	PP904462	Histogenetics, Ossining, USA
C*07:18:08:01	24817840	PQ064106	Dr Luis A Marin, Albacete, Spain
C*07:186	JMDP01K0274	LC831526	Dr Masahiro Satake, Tokyo, Japan
C*07:199:01	JMDP36K0393	LC831527	Dr Masahiro Satake, Tokyo, Japan
C*07:734	71616468	PQ181299	Dr Luis A Marin, Albacete, Spain
C*07:867	HG00026284	MZ967122	Histogenetics, Ossining, USA
C*08:01:01:01	APA	OZ121179	Anthony Nolan Research Institute, London, United Kingdom
C*12:44	JMDP01K0376	LC831528	Dr Masahiro Satake, Tokyo, Japan
C*12:49	JMDP36K0517	LC831529	Dr Masahiro Satake, Tokyo, Japan
C*14:02:01:02	DOP‐ND	OZ121176	Anthony Nolan Research Institute, London, United Kingdom
C*14:24:02	021893951	PP951507	Mrs Ornella Senoussi, La Tronche, France
C*14:45	JMDP36K0501	LC831530	Dr Masahiro Satake, Tokyo, Japan
C*16:112	LIB_5909796	PP996044	Ms Anaregina Araujo, Teresina, Brazil
C*17:49	HG00050007	MH974075	Histogenetics, Ossining, USA
C*18:02:01:01	35020	OZ121127	Anthony Nolan Research Institute, London, United Kingdom
F*01:01:01:08	DBB	OZ079936	Anthony Nolan Research Institute, London, United Kingdom
F*01:01:01:09	APA, COX	OZ079926, OZ121105	Anthony Nolan Research Institute, London, United Kingdom
F*01:01:02:06	SSTO	OZ079963	Anthony Nolan Research Institute, London, United Kingdom
F*01:03:01:01	PGF	OZ079952	Anthony Nolan Research Institute, London, United Kingdom
G*01:01:01:01	DBB	OZ079937	Anthony Nolan Research Institute, London, United Kingdom
G*01:01:01:01	RSA‐ND	OZ121111	Anthony Nolan Research Institute, London, United Kingdom
G*01:01:01:05	PGF	OZ079953	Anthony Nolan Research Institute, London, United Kingdom
G*01:01:02:01	COX	OZ079927	Anthony Nolan Research Institute, London, United Kingdom
G*01:01:02:01	FH13	OZ121109	Anthony Nolan Research Institute, London, United Kingdom
G*01:01:03:03	KWO010 (KAPA)	OZ121110	Anthony Nolan Research Institute, London, United Kingdom
G*01:01:12	SSTO	OZ079964	Anthony Nolan Research Institute, London, United Kingdom
G*01:05:01N	35020	OZ121112	Anthony Nolan Research Institute, London, United Kingdom
G*01:06:01:02	HL‐173	PP996003	Dr Krishna Chaaithanya Itta, Mumbai, India
G*01:19	T‐49	PP659809	Prof Wei Tian, Changsha, China
H*01:01:01:01	DBB	OZ079938	Anthony Nolan Research Institute, London, United Kingdom
H*01:01:01:01	DOP‐ND, AWELLS	OZ121106, OZ121108	Anthony Nolan Research Institute, London, United Kingdom
H*02:01:01:01	COX, BGE	OZ079928, OZ121107	Anthony Nolan Research Institute, London, United Kingdom
H*02:03:01	SSTO	OZ079965	Anthony Nolan Research Institute, London, United Kingdom
H*02:04:01	PGF	OZ079954	Anthony Nolan Research Institute, London, United Kingdom
H*02:05:01:03	35020	OZ121104	Anthony Nolan Research Institute, London, United Kingdom
DRB1*04:205:01	KSR192648	PQ034524	Dr Maria Loginova, Kirov, Russia
DRB1*04:362	KSR174788	PQ034523	Dr Maria Loginova, Kirov, Russia
DRB1*08:03:03	KSR174035	PQ034522	Dr Maria Loginova, Kirov, Russia
DRB1*08:97	021893951	PP951508	Mrs Ornella Senoussi, La Tronche, France
DRB1*09:01:15	457891	PP444720	Prof Edward KL Yang, Hualien, Taiwan
DRB1*11:333	6055632_BLS	PQ009209	Dr Jeane Visentainer, Maringa, Brazil
DRB1*12:110	AMC‐E‐009	PQ015209	Mrs So‐Jung Choi, Seoul, South Korea
DRB1*13:01:43	902080342	PP852895	Mr Thomas Vaudois, Strasbourg, Frances
DRB1*15:01:01:06	AN301263	OZ026116	Anthony Nolan Research Institute, London, United Kingdom
DRB1*16:75	AN303319	OZ038308	Anthony Nolan Research Institute, London, United Kingdom
DRB3*02:171	21‐137‐007986	PQ217225	Dr Qian Hu, Calgary, Canada
DRB3*02:205	JH0129	PQ137838	Dr Maria Bettinotti, Baltimore, USA
DRB4*01:03:01:05	PSCDA0442	PP998052	Dr Seik‐Soon Khor, Tokyo, Japan
DRB4*01:03:01:08	PSCDA0423	PP998053	Dr Seik‐Soon Khor, Tokyo, Japan
DRB4*01:03:02:01	PSCDA1100	PP998054	Dr Seik‐Soon Khor, Tokyo, Japan
DRB4*01:138	78820724835	PQ043872	Dr Cathi Murphey, San Antonio, USA
DRB5*01:01:01:02	PSCDA1150	PP977511	Dr Seik‐Soon Khor, Tokyo, Japan
DRB5*01:139	24011857126HS28053	PP887437	Dr Cathi Murphey, San Antonio, USA
DRB5*01:140	22‐144‐014597	PQ217226	Dr Qian Hu, Calgary, Canada
DRB5*02:02:01	PSCDA1064	PP998051	Dr Seik‐Soon Khor, Tokyo, Japan
DQA1*01:02:21	JH0143	PQ196104	Dr Maria Bettinotti, Baltimore, USA
DQA1*01:02:26	509713	PQ058602	Mr Alessandro Pirri, Curitiba, Brazil
DQA1*01:05:05	JH0139	PQ196101	Dr Maria Bettinotti, Baltimore, USA
DQA1*01:125	HG00050329	ON987374	Histogenetics, Ossining, USA
DQA1*03:01:01:06	UERJ‐C20247	PQ066376	Dr Romulo Vianna, Rio de Janeiro, Brazil
DQA1*03:03:09	HG00050328	ON987373	Histogenetics, Ossining, USA
DQA1*04:01:04	HG00050330	ON987375	Histogenetics, Ossining, USA
DQA1*04:08	021888809	PP951506	Mrs Ornella Senoussi, La Tronche, France
DQA1*05:01:16	61022351	PP922135	Dr Dolores Planelles, Valencia, Spain
DQB1*05:02:01:01	201‐00513	PP932466	Ms Yuanli Zhao, Xi'an, China
DQB1*05:02:01:05	201‐00611	PP951444	Ms Yuanli Zhao, Xi'an, China
DQB1*05:02:01:17	GR‐EVA‐53106	PP936201	Dr Diamanto Kouniaki, Athens, Greece
DQB1*05:03:01:10	DKMS‐LSL_ID21327146	OZ053390	DKMS Life Sciences Lab., Dresden, Germany
DQB1*05:03:02	DKMS‐LSL_ID19981529	OZ053327	DKMS Life Sciences Lab., Dresden, Germany
DQB1*05:03:02	DKMS‐LSL_ID21508352	OZ053366	DKMS Life Sciences Lab., Dresden, Germany
DQB1*05:300	DKMS‐LSL_ID21376610	OZ053380	DKMS Life Sciences Lab., Dresden, Germany
DQB1*05:312	DKMS‐LSL_ID21138057	OZ053351	DKMS Life Sciences Lab., Dresden, Germany
DQB1*06:02:01:40	GR‐EVA‐53090	PP936202	Dr Diamanto Kouniaki, Athens, Greece
DQB1*06:03:49	DKMS‐LSL_ID21098751	OZ053346	DKMS Life Sciences Lab., Dresden, Germany
DQB1*06:09:09	71616486	PQ181301	Dr Luis A Marin, Albacete, Spain
DQB1*06:237	DQ0607020023	PQ094466	Dr Justine Schmitt, Liege, Belgium
DQB1*06:273	71616472	PQ181300	Dr Luis A Marin, Albacete, Spain
DQB1*06:333	KSR168569	PQ034518	Dr Maria Loginova, Kirov, Russia
DQB1*06:409	DKMS‐LSL_ID20826775	OZ053316	DKMS Life Sciences Lab., Dresden, Germany
DQB1*06:421	KSR173107	PQ034520	Dr Maria Loginova, Kirov, Russia
DQB1*06:424	DKMS‐LSL_ID21424063	OZ053377	DKMS Life Sciences Lab., Dresden, Germany
DQB1*06:424	DKMS‐LSL_ID20852248	OZ053336	DKMS Life Sciences Lab., Dresden, Germany
DQB1*06:431	DKMS‐LSL_ID21366706	OZ053382	DKMS Life Sciences Lab., Dresden, Germany
DQB1*06:460	DKMS‐LSL_ID21421709	OZ053378	DKMS Life Sciences Lab., Dresden, Germany
DQB1*06:463	DKMS‐LSL_ID20818824	OZ057430	DKMS Life Sciences Lab., Dresden, Germany
DQB1*06:468	DKMS‐LSL_ID21185384	OZ057506	DKMS Life Sciences Lab., Dresden, Germany
DQB1*02:120	KSR172644	PQ034519	Dr Maria Loginova, Kirov, Russia
DQB1*02:213	KSR191705	PQ034521	Dr Maria Loginova, Kirov, Russia
DQB1*02:220	DKMS‐LSL_ID20862937	OZ053337	DKMS Life Sciences Lab., Dresden, Germany
DQB1*02:223	901664995	PP868643	Mr Thomas Vaudois, Strasbourg, Frances
DQB1*02:223	DKMS‐LSL_ID21046115	OZ057514	DKMS Life Sciences Lab., Dresden, Germany
DQB1*02:224	DKMS‐LSL_ID21527779	OZ057439	DKMS Life Sciences Lab., Dresden, Germany
DQB1*03:01:57	DKMS‐LSL_ID21568312	OZ057477	DKMS Life Sciences Lab., Dresden, Germany
DQB1*03:36	DKMS‐LSL_ID21174023	OZ053355	DKMS Life Sciences Lab., Dresden, Germany
DQB1*03:309:02	DKMS‐LSL_ID20902612	OZ057459	DKMS Life Sciences Lab., Dresden, Germany
DQB1*03:309:02	DKMS‐LSL_ID21338879	OZ053387	DKMS Life Sciences Lab., Dresden, Germany
DQB1*03:309:02	DKMS‐LSL_ID21323260	OZ053391	DKMS Life Sciences Lab., Dresden, Germany
DQB1*03:361:02	RP‐285921	PP989836	Dr Neifi Hassan Saloum Deghaide, Ribeirao Preto, Brazil
DQB1*03:476	DKMS‐LSL_ID21466259	OZ057489	DKMS Life Sciences Lab., Dresden, Germany
DQB1*03:478	DKMS‐LSL_ID21076683	OZ057510	DKMS Life Sciences Lab., Dresden, Germany
DQB1*03:496	DKMS‐LSL_ID20998523	OZ057432	DKMS Life Sciences Lab., Dresden, Germany
DQB1*03:502	DKMS‐LSL_ID21340744	OZ053386	DKMS Life Sciences Lab., Dresden, Germany
DQB1*03:502	DKMS‐LSL_ID21564813	OZ053363	DKMS Life Sciences Lab., Dresden, Germany
DQB1*03:502	DKMS‐LSL_ID20746498	OZ053329	DKMS Life Sciences Lab., Dresden, Germany
DQB1*03:504	DKMS‐LSL_ID21128278	OZ053350	DKMS Life Sciences Lab., Dresden, Germany
DQB1*03:505	DKMS‐LSL_ID20579991	OZ057445	DKMS Life Sciences Lab., Dresden, Germany
DQB1*03:523	509649	PQ041961	Mr Alessandro Pirri, Curitiba, Brazil
DQB1*04:01:01:03	201‐00533	PP935220	Ms Yuanli Zhao, Xi'an, China
DQB1*04:02:01:04	201‐00543	PP938691	Ms Yuanli Zhao, Xi'an, China
DPA1*01:03:29	HG00031573	OL419342	Histogenetics, Ossining, USA
DPA1*01:69	HG00050312	ON987392	Histogenetics, Ossining, USA
DPA1*01:192	FSIMM_IMM‐24‐27906	OZ077747	Clinical Immunology, PathWest, Perth, Australia
DPA1*02:01:01:07	201‐00346	PP961272	Ms Yuanli Zhao, Xi'an, China
DPA1*02:01:01:12	201_01324	PP961282	Ms Yuanli Zhao, Xi'an, China
DPA1*02:02:02:07	201‐00377	PP961273	Ms Yuanli Zhao, Xi'an, China
DPA1*02:03:06	incabrp0413	PP992534	Mr Vinicius Stelet, Rio de Janeiro, Brazil
DPA1*02:50:01	HG00031587	OL419356	Histogenetics, Ossining, USA
DPA1*02:143	SMC014	LC833328	Dr Eunsuk Kang, Seoul, South Korea
DPA1*03:18	JH0146	PQ202988	Dr Maria Bettinotti, Baltimore, USA
DPB1*01:01:11	DKMS‐LSL_ID20766307	OZ123260	DKMS Life Sciences Lab., Dresden, Germany
DPB1*02:01:02:21	DKMS‐LSL_ID20642876	OZ125927	DKMS Life Sciences Lab., Dresden, Germany
DPB1*02:01:02:52	AN303061	OZ026225	Anthony Nolan Research Institute, London, United Kingdom
DPB1*02:01:02:92	DKMS‐LSL_ID20649510	OZ125930	DKMS Life Sciences Lab., Dresden, Germany
DPB1*02:01:02:100	DKMS‐LSL_ID21422039	OZ125959	DKMS Life Sciences Lab., Dresden, Germany
DPB1*02:01:62	DKMS‐LSL_ID21147425	OZ123333	DKMS Life Sciences Lab., Dresden, Germany
DPB1*02:01:64	SZ‐231	PP831188	Dr Hong‐Yan Zou, Shenzhen, China
DPB1*02:01:68	DKMS‐LSL_ID21007716	OZ125952	DKMS Life Sciences Lab., Dresden, Germany
DPB1*03:01:01:26	DKMS‐LSL_ID21436333	OZ123319	DKMS Life Sciences Lab., Dresden, Germany
DPB1*04:01:01:101	DKMS‐LSL_ID20726278	OZ125939	DKMS Life Sciences Lab., Dresden, Germany
DPB1*04:01:01:107	DKMS‐LSL_ID20739845	OZ125940	DKMS Life Sciences Lab., Dresden, Germany
DPB1*04:01:01:110	DKMS‐LSL_ID20561207	OZ123255	DKMS Life Sciences Lab., Dresden, Germany
DPB1*04:01:01:150	AN303318	OZ038306	Anthony Nolan Research Institute, London, United Kingdom
DPB1*04:02:26	DKMS‐LSL_ID21129646	OZ123334	DKMS Life Sciences Lab., Dresden, Germany
DPB1*104:01:07	DKMS‐LSL_ID20642876	OZ125928	DKMS Life Sciences Lab., Dresden, Germany
DPB1*104:01:08	901139980	PP852898	Mr Thomas Vaudois, Strasbourg, Frances
DPB1*105:01:01:12	DKMS‐LSL_ID21639086	OZ123293	DKMS Life Sciences Lab., Dresden, Germany
DPB1*246:01	LIB_6152843	PP996041	Ms Anaregina Araujo, Teresina, Brazil
DPB1*1344:01:02	DKMS‐LSL_ID21059136	OZ123283	DKMS Life Sciences Lab., Dresden, Germany
DPB1*1363:01	DKMS‐LSL_ID21341415	OZ125967	DKMS Life Sciences Lab., Dresden, Germany
DPB1*1391:01	DKMS‐LSL_ID21309705	OZ125971	DKMS Life Sciences Lab., Dresden, Germany
DPB1*1410:01	DKMS‐LSL_ID21259607	OZ123328	DKMS Life Sciences Lab., Dresden, Germany
DPB1*1463:01N	LIB_5989553	PP996042	Ms Anaregina Araujo, Teresina, Brazil
DPB1*1493:01	G230506004	OZ060927	Miss Xingjie Li, Chengdu, China
DPB1*1548:01N	DKMS‐LSL_ID20940453	OZ123278	DKMS Life Sciences Lab., Dresden, Germany
DPB1*1555:01	DKMS‐LSL_ID21044818	OZ125983	DKMS Life Sciences Lab., Dresden, Germany
DPB1*1591:01	HG00052236	PP926163	Histogenetics, Ossining, USA
DPB1*1594:01	DKMS‐LSL_ID21100161	OZ123286	DKMS Life Sciences Lab., Dresden, Germany
DPB1*1621:01	DKMS‐LSL_ID20580290	OZ125917	DKMS Life Sciences Lab., Dresden, Germany
DPB1*1626:01Q	103926341	PP951066	Dr Antonio Balas, Madrid, Spain
DMA*01:01:01:02	SSTO, COX	OZ079922, OZ079959	Anthony Nolan Research Institute, London, United Kingdom
DMA*01:01:01:03	PGF	OZ079948	Anthony Nolan Research Institute, London, United Kingdom
DMA*01:02:01:01	DBB	OZ079932	Anthony Nolan Research Institute, London, United Kingdom
DMB*01:01:01:02	DBB	OZ079933	Anthony Nolan Research Institute, London, United Kingdom
DMB*01:03:01:01	SSTO	OZ079960	Anthony Nolan Research Institute, London, United Kingdom
DMB*01:03:01:03	PGF	OZ079949	Anthony Nolan Research Institute, London, United Kingdom
DMB*01:03:01:04	COX	OZ079923	Anthony Nolan Research Institute, London, United Kingdom
DOA*01:01:01:01	DBB	OZ079934	Anthony Nolan Research Institute, London, United Kingdom
DOA*01:01:02:02	PGF	OZ079950	Anthony Nolan Research Institute, London, United Kingdom
DOA*01:01:04:01	SSTO	OZ079961	Anthony Nolan Research Institute, London, United Kingdom
DOA*01:01:05	COX	OZ079924	Anthony Nolan Research Institute, London, United Kingdom
DOB*01:01:01:01	PGF	OZ079951	Anthony Nolan Research Institute, London, United Kingdom
DOB*01:01:01:04	SSTO	OZ079962	Anthony Nolan Research Institute, London, United Kingdom
DOB*01:01:03:02	COX	OZ079925	Anthony Nolan Research Institute, London, United Kingdom
DOB*01:05	DBB	OZ079935	Anthony Nolan Research Institute, London, United Kingdom
MICB*054	24011857126HS28053	PP887440	Dr Cathi Murphey, San Antonio, USA

**TABLE 3:** Recently published sequences.

HLA allele	References
A*01:01:148	[7]
A*01:459	[8]
A*02:01:189	[9]
A*02:03:01:06Q	[7]
A*02:06:34	[8]
A*02:947:02	[10]
A*02:1134	[11]
A*02:1135	[12]
A*02:1144	[13]
A*02:1146	[14]
A*02:1174	[15]
A*03:478	[16]
A*11:01:01:86	[7]
A*11:01:117	[17]
A*11:01:127	[18]
A*11:127N	[19]
A*11:159	[20]
A*23:140	[21]
A*24:140	[20]
A*24:196	[20]
A*24:284	[20]
A*24:393	[22]
A*24:617	[23]
A*24:618	[24]
A*24:630	[25]
A*24:632	[26]
A*26:247	[27]
A*30:221	[28]
A*31:232N	[29]
A*32:01:56	[30]
A*33:01:21	[31]
A*33:33:02	[32]
A*33:73N	[20]
A*68:03:04	[11]
A*68:115	[20]
B*07:510	[33]
B*13:194	[34]
B*15:689	[35]
B*15:694	[34]
B*15:696	[15]
B*15:699	[36]
B*18:37:03	[37]
B*18:243	[38]
B*18:244	[29]
B*27:276	[39]
B*35:03:39	[15]
B*35:235	[20]
B*35:603	[35]
B*35:611	[40]
B*37:109N	[7]
B*38:01:01:18	[41]
B*38:02:12	[11]
B*38:118	[29]
B*39:204	[11]
B*40:86	[42]
B*40:400	[43]
B*40:510N	[44]
B*40:540N	[45]
B*40:571N	[7]
B*41:84	[46]
B*46:96	[47]
B*49:01:01:22	[48]
B*49:01:25	[35]
B*51:01:44	[49]
B*51:197	[50]
B*51:360	[51]
B*51:411	[52]
B*51:413	[53]
B*53:01:32	[11]
B*56:100	[54]
B*58:147	[7]
C*01:02:01:74Q	[7]
C*01:273	[55]
C*01:274	[56]
C*02:240Q	[15]
C*02:243	[15]
C*02:246	[57]
C*03:03:71	[7]
C*03:100	[20]
C*03:677	[36]
C*03:683	[29]
C*04:01:01:186	[58]
C*04:32:01:02	[59]
C*05:01:81	[60]
C*05:01:82	[15]
C*06:387	[29]
C*07:01:01:141	[61]
C*07:01:01:142	[59]
C*07:02:81	[62]
C*07:02:151	[63]
C*07:370	[20]
C*07:1015	[11]
C*07:1132	[64]
C*07:1148	[29]
C*08:04:01:05	[59]
C*08:271	[11]
C*08:291	[65]
C*12:03:01:68	[59]
C*12:03:01:69	[59]
C*12:03:89	[15]
C*12:109	[20]
C*14:02:01:31	[66]
C*14:163	[15]
C*15:42	[20]
C*15:68	[20]
C*15:279	[67]
C*16:01:01:41	[59]
C*17:78	[68]
C*18:20	[69]
F*01:12	[70]
G*01:01:01:34	[71]
G*01:19	[72]
G*01:46	[73]
G*01:55	[74]
DRB1*03:215	[75]
DRB1*04:379N	[76]
DRB1*08:126	[77]
DRB1*09:54	[8]
DRB1*13:357	[78]
DRB3*02:171	[79]
DRB3*02:202	[80]
DRB3*02:209	[81]
DRB3*03:69	[82]
DRB4*01:179	[10]
DRB4*01:182	[83]
DRB5*01:140	[79]
DQA1*01:02:24	[84]
DQA1*02:01:11	[11]
DQA1*03:75	[85]
DQA1*03:79	[86]
DQA1*05:03:03	[87]
DQA1*05:112	[88]
DQA1*05:115	[89]
DQB1*05:01:50	[90]
DQB1*05:305	[91]
DQB1*06:02:01:40	[92]
DQB1*06:02:61	[93]
DQB1*06:02:62	[94]
DQB1*06:486	[90]
DQB1*02:211	[95]
DQB1*03:01:01:60	[96]
DQB1*03:01:01:61	[96]
DQB1*03:01:01:62	[96]
DQB1*03:01:01:63	[96]
DQB1*03:01:01:64	[97]
DQB1*03:01:61	[98]
DQB1*03:02:01:23	[96]
DQB1*03:02:01:24	[96]
DQB1*03:02:01:25	[96]
DQB1*03:03:02:14	[96]
DQB1*03:14:02	[20]
DQB1*03:517	[99]
DQB1*03:522	[7]
DQB1*03:525	[78]
DQB1*04:100	[29]
DPA1*01:03:01:73	[100]
DPA1*01:03:01:80	[100]
DPA1*01:03:01:82	[100]
DPA1*01:03:38:02	[11]
DPA1*01:12:03	[101]
DPA1*01:104	[11]
DPA1*01:155:01:02	[100]
DPA1*02:02:02:16	[100]
DPA1*02:134	[102]
DPA1*02:139	[103]
DPA1*02:140N	[103]
DPB1*04:02:01:44	[104]
DPB1*04:02:24	[105]
DPB1*14:01:15	[106]
DPB1*21:01:02	[107]
DPB1*1553:01	[108]
DPB1*1618:01	[109]
MICA*112:02	[110]
MICB*002:06	[111]
MICB*005:15	[112]
MICB*055	[112]

All new and confirmatory sequences should now be submitted directly to the WHO Nomenclature Committee for Factors of the HLA System via the IPD‐IMGT/HLA Database using the sequence submission tool provided [5, 6]. The IPD‐IMGT/HLA Database may be accessed via the World Wide Web at: www.ebi.ac.uk/ipd/imgt/hla.

## Conflicts of Interest

The authors declare no conflicts of interest.

## References

[1] S. G. E. Marsh , “Nomenclature for Factors of the HLA System, Update April, May and June 2024,” HLA 104, no. 1 (July 2024): e15614, 10.1111/tan.15614. 39040002

[2] S. G. E. Marsh , E. D. Albert , W. F. Bodmer , et al., “Nomenclature for Factors of the HLA System, 2010,” Tissue Antigens 75, no. 4 (April 2010): 291–455, 10.1111/j.1399-0039.2010.01466.x. 20356336 PMC2848993

[3] A. J. E. French , J. A. M. Lucas , M. A. Cooper , S. G. E. Marsh , and N. P. Mayor , “Identification of 43 Novel HLA Alleles by PacBio Single Molecule Real‐Time Sequencing in Haematopoietic Cell Donors,” HLA 104, no. 2 (August 2024): e15645, 10.1111/tan.15645. 39148299

[4] J. A. M. Lucas , S. J. F. Hopper , J. Robinson , S. G. E. Marsh , and N. P. Mayor , “60 HLA Class I and 115 HLA Class II Sequence Confirmations Submitted to the IPD‐IMGT/HLA Database,” HLA 104, no. 1 (July 2024): e15607, 10.1111/tan.15607. 39073257

[5] D. J. Barker , G. Maccari , X. Georgiou , et al., “The IPD‐IMGT/HLA Database,” Nucleic Acids Research 51, no. D1 (January 2023): D1053–D1060, 10.1093/nar/gkac1011. 36350643 PMC9825470

[6] J. Robinson , D. J. Barker , and S. G. E. Marsh , “25 Years of the IPD‐IMGT/HLA Database,” HLA 103, no. 6 (June 2024): e15549, 10.1111/tan.15549. 38936817

[7] S. Archita , P. S. Shruthi , A. Deepika , K. Manpreet , and P. I. Megha , “Identification of Nine Novel HLA Alleles by Next‐Generation Sequencing in Individuals From India,” HLA 104, no. 3 (September 2024): e15676, 10.1111/tan.15676. 39234804

[8] S. Zhao , C. Chen , F. Wang , W. Zhang , and F. Zhu , “The Novel HLA‐DRB1 Allele, HLA‐DRB1*09:54 Was Identified in a Chinese Individual,” HLA 104, no. 1 (July 2024): e15596, 10.1111/tan.15596. 38993157

[9] M. Troiano , A. G. Bianculli , A. Di Luzio , R. M. Pinto , and M. Andreani , “Identification of the Novel HLA‐A*02:01:189 Allele,” HLA 104, no. 2 (August 2024): e15651, 10.1111/tan.15651. 39192552

[10] W. D. Wu , X. X. Zhong , Z. R. Quan , S. H. Luo , and R. Yuan , “A Novel HLA‐DRB4 Allele, HLA‐DRB4*01:179,” HLA 104, no. 1 (July 2024): e15600, 10.1111/tan.15600. 39015081

[11] M. D. Gautreaux , E. F. O'Shields , E. M. Netherton , et al., “Identification of Novel HLA Alleles Discovered in 2022‐2023,” Human Immunology 85, no. 3 (May 2024): 110772, 10.1016/j.humimm.2024.110772. 38461131

[12] V. Byers , H. Roh , C. Zheng , J. Ge , and S. Y. Choo , “The Novel HLA‐A*02:1135 Allele Identified in a Chinese Individual From new York City,” HLA 104, no. 2 (August 2024): e15602, 10.1111/tan.15602. 39132697

[13] Y. P. Xu , F. Yang , W. X. Xia , and S. Q. Gao , “Identification of the Novel Allele HLA‐A*02:1144 in a Chinese Platelet Donor,” HLA 104, no. 1 (July 2024): e15612, 10.1111/tan.15612. 39041339

[14] Y. Xu , Y. P. Xie , L. An , Y. M. Li , and P. F. Shi , “The Novel HLA‐A*02:1146 Allele, Identified by Sanger Dideoxy Nucleotide Sequencing in a Chinese Individual,” HLA 104, no. 2 (August 2024): e15576, 10.1111/tan.15576. 39149763

[15] M. Loginova , O. Makhova , D. Smirnova , and I. Paramonov , “Identification of Eight New HLA Class I Alleles by Next‐Generation Sequencing,” HLA 104, no. 1 (July 2024): e15604, 10.1111/tan.15604. 39015089

[16] J. A. Kim , H. I. Bang , S. Chae , H. Y. Im , and J. W. Shin , “The Novel HLA‐A Allele, HLA‐A*03:478, Identified in a Korean Individual,” HLA 104, no. 1 (July 2024): e15595, 10.1111/tan.15595. 38993156

[17] Y. He , Q. Li , F. Wang , C. Chen , and F. Zhu , “Identification of the Novel HLA‐A*11:01:117 Allele by Next‐Generation Sequencing,” HLA 104, no. 1 (July 2024): e15608, 10.1111/tan.15608. 39041330

[18] Q. X. Lin , G. B. Zhang , L. An , Y. M. Li , and T. Yang , “The Novel HLA‐A*11:01:127 Allele, Identified by Sanger Dideoxy Nucleotide Sequencing in a Chinese Individual,” HLA 103, no. 6 (June 2024): e15564, 10.1111/tan.15564. 38923824

[19] K. L. Yang and P. Y. Lin , “Detection of the HLA‐A*11:127N Allele in a Taiwanese Individual,” HLA 103, no. 6 (June 2024): e15578, 10.1111/tan.15578. 38923289

[20] P. I. M. Vinod , P. S. Shruthi , A. Sharma , D. Arora , and M. Kour , “Extending HLA Allele Sequences Using Next‐Generation Sequencing Technology,” HLA 104, no. 2 (August 2024): e15656, 10.1111/tan.15656. 39189248

[21] A. Pirri , R. Slowik , B. M. da Graca , and P. S. de Araujo‐Souza , “The Novel HLA‐A*23:140 Allele Was Identified in a Brazilian Bone Marrow Volunteer Donor,” HLA 104, no. 3 (September 2024): e15661, 10.1111/tan.15661. 39234787

[22] C. Y. Lin and C. L. Chang , “The Novel HLA‐A*24:393 Allele, Identified by Sanger Dideoxy Nucleotide Sequencing in a Taiwanese Individual,” HLA 104, no. 3 (September 2024): e15690, 10.1111/tan.15690. 39291368

[23] S. Q. Feng , W. Fan , L. An , Y. M. Li , and R. Liu , “The Novel HLA‐A*24:617 Allele, Identified by Sanger Dideoxy Nucleotide Sequencing in a Chinese Individual,” HLA 104, no. 2 (August 2024): e15559, 10.1111/tan.15559. 39148264

[24] C. Zheng , V. Eravelli , M. Kosierb , J. Ge , and S. Y. Choo , “The Novel HLA‐A*24:618 Allele Identified in Ecuadorian Individual From New York City,” HLA 104, no. 2 (August 2024): e15662, 10.1111/tan.15662. 39189315

[25] L. Pan , A. Zhang , L. Zhao , W. Tang , and B. Fu , “Characterisation of the Novel HLA‐A*24:630 Allele by Sequencing‐Based Typing,” HLA 104, no. 2 (August 2024): e15650, 10.1111/tan.15650. 39148305

[26] P. Mantzios , T. Athanassiades , P. Mantziou , V. Kitsiou , and A. Tsirogianni , “Identification of the New Allele HLA‐A*24:632 in a Greek Individual Using Next Generation Sequencing,” HLA 104, no. 1 (July 2024): e15622, 10.1111/tan.15622. 39041361

[27] M. Cargou , V. Elsermans , I. Top , G. Guidicelli , and J. Visentin , “Characterisation of the Novel HLA‐A*26:247 Allele by Sequencing‐Based Typing,” HLA 104, no. 3 (September 2024): e15673, 10.1111/tan.15673. 39225190

[28] T. Galluccio , P. Giustiniani , A. Di Luzio , G. Testa , and M. Andreani , “Identification of the Novel HLA‐A*30:221 Allele by Next‐Generation Sequencing,” HLA 104, no. 1 (July 2024): e15592, 10.1111/tan.15592. 38982852

[29] M. Loginova , O. Makhova , and I. Paramonov , “Seven New HLA Alleles Characterised by Next‐Generation Sequencing,” HLA 104, no. 3 (September 2024): e15677, 10.1111/tan.15677. 39234850

[30] B. Rhodes‐Clark and C. Redding , “The Novel HLA‐A*32 Allele, HLA‐A*32:01:56, Identified by Next Generation Sequencing,” HLA 104, no. 3 (September 2024): e15693, 10.1111/tan.15693. 39291360

[31] M. Cunha , G. Andrade , R. Vianna , L. C. Porto , and D. Secco , “Characterisation of the Novel HLA‐A*33:01:21 Allele, First Identified in a Brazilian Individual,” HLA 104, no. 1 (July 2024): e15618, 10.1111/tan.15618. 39056465

[32] S. Agarwal , S. Kumari , N. Jaiswal , V. Kumar , and R. Kumar , “Characterisation of a Novel HLA‐A*33:33:02 Allele in Two Members of a North Indian Family,” HLA 104, no. 2 (August 2024): e15643, 10.1111/tan.15643. 39132701

[33] M. Loginova , N. Morozova , and I. Paramonov , “HLA‐B*07:510, a Novel HLA‐B Allele Identified in Two Not Relative Individuals,” HLA 104, no. 3 (September 2024): e15699, 10.1111/tan.15699. 39291352

[34] B. Valatkaite‐Rakstiene and A. Jakubauskas , “Characterisation of Two Novel HLA‐B Alleles, B*13:194 and B*15:694 in Individuals From Lithuania,” HLA 104, no. 3 (September 2024): e15681, 10.1111/tan.15681. 39234823

[35] M. Loginova and I. Paramonov , “Description of Three New HLA‐B Alleles: HLA‐B*15:689, HLA‐B*35:603 and HLA‐B*49:01:25,” HLA 104, no. 2 (August 2024): e15638, 10.1111/tan.15638. 39091265

[36] M. Norgaard , L. O. Drechsler , and B. K. Moller , “Characterisation of the Novel HLA‐B*15:699 and HLA‐C*03:677 Alleles Identified in Danish Individuals,” HLA 104, no. 3 (September 2024): e15701, 10.1111/tan.15701. 39307952

[37] J. Pinto , V. Oliveira , R. Vianna , L. C. Porto , and D. Secco , “Characterisation of the Novel HLA‐B*18:37:03 Allele, First Identified in a Brazilian Individual,” HLA 104, no. 3 (September 2024): e15668, 10.1111/tan.15668. 39225154

[38] D. Secco , M. Cunha , G. Andrade , R. Vianna , and L. C. Porto , “Characterisation of the Novel HLA‐B*18:243 Allele Using Short‐ and Long‐Read Sequencing Technologies,” HLA 103, no. 6 (June 2024): e15579, 10.1111/tan.15579. 38923223

[39] G. Tiziana , G. Paola , G. Annalisa , B. Francesca , and A. Marco , “Identification of the Novel HLA‐B*27:276 Allele by Next‐Generation Sequencing,” HLA 104, no. 1 (July 2024): e15605, 10.1111/tan.15605. 39054740

[40] M. Loginova , D. Smirnova , and I. Paramonov , “Discovery of the Novel HLA‐B*35:611 Allele, a Variant of the HLA‐B*35:64:01 Allele,” HLA 104, no. 3 (September 2024): e15703, 10.1111/tan.15703. 39291394

[41] D. Kouniaki , V. Kitsiou , and A. Tsirogianni , “Characterisation of the Novel HLA‐B*38:01:01:18 Allele by Next‐Generation Sequencing,” HLA 103, no. 6 (June 2024): e15573, 10.1111/tan.15573. 38923829

[42] S. Q. Gao , W. X. Xia , F. Yang , H. Zhang , and Y. P. Xu , “Genomic Full‐Length Sequence of the HLA‐B*40:86 Allele Identified in a Chinese Individual,” HLA 103, no. 6 (June 2024): e15491, 10.1111/tan.15491. 38936820

[43] K. L. Yang and P. Y. Lin , “Recognition of the HLA‐B*40:400 Allele and Its Associated HLA Haplotype in a Taiwanese Individual,” HLA 103, no. 6 (June 2024): e15585, 10.1111/tan.15585. 38934046

[44] X. X. Zhong and Z. R. Quan , “A Novel Null HLA‐B Allele, B*40:510N, due to a Single Nucleotide Deletion in Exon 2,” HLA 104, no. 3 (September 2024): e15684, 10.1111/tan.15684. 39279448

[45] J. J. Yang and H. B. Oh , “Genomic Full‐Length Sequence of the Novel HLA‐B*40:540N Allele,” HLA 104, no. 2 (August 2024): e15652, 10.1111/tan.15652. 39148314

[46] A. Sharma , S. Al Agbar , D. Beaune , and A. M. E. Sidahmed , “The Newly Characterised HLA‐B*41:84 Allele, Found in a Prospective Liver Transplant Candidate,” HLA 104, no. 2 (August 2024): e15582, 10.1111/tan.15582. 39189316

[47] L. Dong , W. Pi , N. Chen , W. Zhang , and F. Zhu , “Characterisation of the Novel HLA‐B*46:96 Allele by Next‐Generation Sequencing,” HLA 104, no. 1 (July 2024): e15593, 10.1111/tan.15593. 38982856

[48] R. M. Jaiswal , S. Swaroop , A. Singla , S. Sharma , and S. Godara , “Identification of the Novel HLA‐B*49:01:01:22 Allele in an Indian Renal Transplant Donor by Next Generation Sequencing,” HLA 104, no. 3 (September 2024): e15665, 10.1111/tan.15665. 39227306

[49] K. L. Yang and P. Y. Lin , “Detection of the HLA‐B*51:01:44 Allele in a Taiwanese Individual,” HLA 104, no. 3 (September 2024): e15694, 10.1111/tan.15694. 39307922

[50] N. Kouprie , L. B. Bungener , A. J. A. Lambeck , B. J. Kroesen , and B. G. Hepkema , “HLA‐B*51:197: A Composite Product of Interlocus Recombination of B*51:01:01:01 and C*01:02:01:01,” HLA 104, no. 1 (July 2024): e15606, 10.1111/tan.15606. 39041326

[51] L. Dong , Q. Li , F. Wang , W. Zhang , and F. Zhu , “Identification of the Novel HLA‐B*51:360 Allele in a Chinese Individual,” HLA 104, no. 2 (August 2024): e15660, 10.1111/tan.15660. 39189310

[52] M. Cargou , V. Elsermans , I. Top , G. Guidicelli , and J. Visentin , “Characterisation of the Novel HLA‐B*51:411 Allele by Sequencing‐Based Typing,” HLA 104, no. 3 (September 2024): e15679, 10.1111/tan.15679. 39234810

[53] Q. Pan , X. Ma , Y. You , Y. Li , and X. Zhou , “Identification of the Novel HLA‐B*51:413 Allele by Next‐Generation Sequencing in a Chinese Han Individual,” HLA 104, no. 1 (July 2024): e15617, 10.1111/tan.15617. 39056461

[54] M. Lepage , X. Fournel , I. Favre‐Victoire , P. Moskovtchenko , and V. Dubois , “Identification of the Novel HLA‐B*56:100 Allele by Next‐Generation Sequencing,” HLA 104, no. 3 (September 2024): e15691, 10.1111/tan.15691. 39291331

[55] D. Secco , M. Cunha , G. Andrade , R. Vianna , and L. C. Porto , “Characterisation of the Novel HLA‐C*01:273 Allele, First Identified in a Brazilian Individual,” HLA 104, no. 1 (July 2024): e15591, 10.1111/tan.15591. 38982842

[56] X. Lian , L. J. Wang , Y. J. Jia , and T. C. Sun , “The Novel HLA‐C*01:274 Allele, Identified by Sanger Dideoxy Nucleotide Sequencing in a Chinese Individual,” HLA 104, no. 1 (July 2024): e15620, 10.1111/tan.15620. 39073028

[57] X. M. Shi and S. J. Gao , “Identification of a Novel HLA‐C Allele, HLA‐C*02:246,” HLA 104, no. 1 (July 2024): e15629, 10.1111/tan.15629. 39073238

[58] P. Mantzios , T. Athanassiades , D. Kouniaki , V. Kitsiou , and A. Tsirogianni , “Identification of the New Allele HLA‐C*04:01:01:186 in a Greek Individual Using Next Generation Sequencing,” HLA 104, no. 1 (July 2024): e15569, 10.1111/tan.15569. 39016219

[59] M. R. Moya‐Quiles and M. Muro , “Identification of Six New HLA‐C Variants With Nucleotide Changes in Non‐Coding Regions,” HLA 104, no. 3 (September 2024): e15692, 10.1111/tan.15692. 39291365

[60] S. Gonen and C. Demir , “Identification of a New HLA‐C Allele, HLA‐C*05:01:81,” HLA 103, no. 6 (June 2024): e15552, 10.1111/tan.15552. 38923200

[61] D. Kouniaki , T. Athanassiades , and A. Tsirogianni , “A Novel HLA‐C*07 Variant Allele, HLA‐C*07:01:01:141, Identified by Next‐Generation Sequencing,” HLA 104, no. 3 (September 2024): e15687, 10.1111/tan.15687. 39291294

[62] C. L. Chang and C. Y. Lin , “The Novel HLA Class I Allele HLA‐C*07:02:81 Differs From HLA‐C*07:02:01:01 by a Synonymous Mutation,” HLA 104, no. 3 (September 2024): e15672, 10.1111/tan.15672. 39234798

[63] V. Elsermans , M. Cargou , T. Pajot , I. Top , and M. Labalette , “Characterisation of the Novel HLA‐C*07:02:151 Allele by Sequencing‐Based Typing,” HLA 104, no. 2 (August 2024): e15635, 10.1111/tan.15635. 39101357

[64] T. Galluccio , R. Cerretti , M. Battarra , P. Giustiniani , and M. Andreani , “Identification of the Novel HLA‐C*07:1132 Allele by Next‐Generation Sequencing,” HLA 104, no. 2 (August 2024): e15641, 10.1111/tan.15641. 39132716

[65] M. R. Moya‐Quiles and M. Muro , “Identification of the Novel HLA‐C*08:291 Allele by Next‐Generation Sequencing,” HLA 104, no. 3 (September 2024): e15689, 10.1111/tan.15689. 39279454

[66] D. Kouniaki and A. Tsirogianni , “The Novel HLA‐C*14:02:01:31 Allele Identified in a Candidate Bone Marrow Donor by Next‐Generation Sequencing,” HLA 104, no. 1 (July 2024): e15598, 10.1111/tan.15598. 38993151

[67] D. M. Li , Y. Y. Jing , Y. J. Jia , and T. C. Sun , “The Novel HLA‐C*15:279 Allele, Identified by Sanger Dideoxy Nucleotide Sequencing in a Chinese Individual,” HLA 104, no. 2 (August 2024): e15631, 10.1111/tan.15631. 39087256

[68] S. Agarwal , S. Kumari , N. Jaiswal , V. Kumar , and R. Kumar , “Identification of Novel HLA‐C*17:78 Allele in North Indian Individuals,” HLA 104, no. 2 (August 2024): e15644, 10.1111/tan.15644. 39132706

[69] N. McLamb , J. E. Jennemann , P. Willey , and C. Liu , “A Novel HLA‐C*18 Variant Allele, HLA‐C*18:20, Identified by Next‐Generation Sequencing,” HLA 104, no. 2 (August 2024): e15647, 10.1111/tan.15647. 39148296

[70] M. Romero , V. N. Stelet , R. Binato , and E. Abdelhay , “The Novel Non‐Classical HLA‐F*01:12 Allele Identified in a Brazilian Individual,” HLA 104, no. 1 (July 2024): e15611, 10.1111/tan.15611. 39039854

[71] Y. Mujumdar , S. V. Thakur , V. M. Bhor , and I. K. Chaaithanya , “HLA‐G*01:01:01:34 Allele Discovered in Two Individuals From a Tribal Community of Western Maharashtra, India,” HLA 103, no. 6 (June 2024): e15587, 10.1111/tan.15587. 38934048

[72] S. W. Yuan , L. Li , and W. Tian , “The Full‐Length Genomic Sequence of the HLA‐G*01:19 Allele,” HLA 104, no. 2 (August 2024): e15654, 10.1111/tan.15654. 39149758

[73] M. Romero , V. N. Stelet , R. Binato , and E. Abdelhay , “The Novel Non‐Classical HLA‐G*01:46 Identified in a Brazilian Individual,” HLA 104, no. 1 (July 2024): e15590, 10.1111/tan.15590. 39015092

[74] Z. R. Quan , J. Liu , B. N. Yang , J. M. Song , and H. Y. Zou , “The Novel Non‐Classical HLA‐G*01:55 Identified in a Chinese Individual,” HLA 104, no. 2 (August 2024): e15657, 10.1111/tan.15657. 39171368

[75] D. Secco , G. Andrade , V. Oliveira , R. Vianna , and L. C. Porto , “Characterisation of the Novel HLA‐DRB1*03:215 Allele Using Short‐ and Long‐Read Sequencing Technologies,” HLA 103, no. 6 (June 2024): e15588, 10.1111/tan.15588. 38934049

[76] L. Blandin , M. Ralazamahaleo , G. Guidicelli , P. Rouzaire , and R. Lemal , “Characterisation of the Novel HLA‐DRB1*04:379N Allele by Sequencing‐Based Typing,” HLA 104, no. 1 (July 2024): e15623, 10.1111/tan.15623. 39054744

[77] V. Elsermans , J. Visentin , T. Pajot , I. Top , and M. Labalette , “Characterisation of the Novel HLA‐DRB1*08:126 Allele by Sequencing‐Based Typing,” HLA 104, no. 2 (August 2024): e15626, 10.1111/tan.15626. 39091257

[78] M. Loginova , D. Smirnova , and I. Paramonov , “Two Novel HLA Class II Alleles Identified by Next‐Generation Sequencing,” HLA 104, no. 2 (August 2024): e15655, 10.1111/tan.15655. 39149765

[79] Q. Hu , A. Abu‐Khader , D. Christian , G. Yang , and N. Berka , “Discovery of Potential Recombinant Alleles HLA‐DRB3*02:171 and HLA‐DRB5*01:140,” HLA 104, no. 3 (September 2024): e15697, 10.1111/tan.15697. 39291436

[80] M. Cargou , M. Andreani , A. G. Bianculli , E. Wojciechowski , and J. Visentin , “Characterisation of the Novel HLA‐DRB3*02:202 Allele by Sequencing‐Based Typing,” HLA 104, no. 3 (September 2024): e15669, 10.1111/tan.15669. 39225183

[81] J. K. Lee , S. Lee , J. W. Park , S. Lim , and E. S. Kang , “Identification of the Novel HLA‐DRB3*02:209 Allele by Next‐Generation Sequencing,” HLA 104, no. 1 (July 2024): e15630, 10.1111/tan.15630. 39073315

[82] I. F. Molinari , A. M. L. de Oliveira , F. P. Massi , B. K. B. Hirata , and J. E. L. Visentainer , “Identification of the Novel HLA‐DRB3*03:69 Allele by Next‐Generation Sequencing,” HLA 104, no. 2 (August 2024): e15646, 10.1111/tan.15646. 39148285

[83] L. Blandin , M. Cargou , E. Wojciechowski , R. Lemal , and P. Rouzaire , “Characterisation of the Novel HLA‐DRB4*01:182 Allele by Sequencing‐Based Typing,” HLA 104, no. 1 (July 2024): e15619, 10.1111/tan.15619. 39073016

[84] M. Cargou , M. Andreani , T. Galluccio , M. Ralazamahaleo , and J. Visentin , “Characterisation of the Novel HLA‐DQA1*01:02:24 Allele by Sequencing‐Based Typing,” HLA 104, no. 3 (September 2024): e15671, 10.1111/tan.15671. 39227182

[85] S. H. Kim , J. J. Yang , and H. B. Oh , “The Novel HLA‐DQA1*03:75 Allele Identified in a Korean Kidney Donor,” HLA 104, no. 1 (July 2024): e15627, 10.1111/tan.15627. 39073250

[86] A. Gaballa , N. Lundin , and T. Eich , “Identification of the Novel HLA‐DQA1*03:79 Allele in a Candidate Bone Marrow Donor by Next Generation Sequencing,” HLA 104, no. 3 (September 2024): e15670, 10.1111/tan.15670. 39225170

[87] M. R. Moya‐Quiles and M. Muro , “Identification of the Novel HLA‐DQA1*05:03:03 Allele by Next‐Generation Sequencing,” HLA 104, no. 1 (July 2024): e15601, 10.1111/tan.15601. 39022880

[88] L. Blandin , J. Visentin , E. Wojciechowski , R. Lemal , and P. Rouzaire , “Characterisation of the Novel HLA‐DQA1*05:112 Allele by Sequencing‐Based Typing,” HLA 104, no. 1 (July 2024): e15621, 10.1111/tan.15621. 39054733

[89] L. A. Marin Rubio and J. Ontanon , “HLA‐DQA1*05:115, the First DQA1 Allele Described With an Alanine at Position 59 of Alpha‐1 Domain,” HLA 104, no. 3 (September 2024): e15695, 10.1111/tan.15695. 39279460

[90] M. Loginova , I. Obukhov , and I. Paramonov , “Two Novel HLA‐DQB1 Alleles Identified by Next Generation Sequencing,” HLA 104, no. 2 (August 2024): e15633, 10.1111/tan.15633. 39091269

[91] N. Chen , Y. He , L. Dong , W. Zhang , and F. Zhu , “Characterisation of the Novel HLA‐DQB1*05:305 Allele Using Next‐Generation Sequencing,” HLA 104, no. 3 (September 2024): e15667, 10.1111/tan.15667. 39225161

[92] D. Kouniaki , K. Tarassi , and A. Tsirogianni , “A Novel HLA‐DQB1*06 Variant Allele, HLA‐DQB1*06:02:01:40, Identified by Next‐Generation Sequencing,” HLA 104, no. 3 (September 2024): e15706, 10.1111/tan.15706. 39311807

[93] A. G. Bianculli , F. Besi , M. Troiano , P. Giustiniani , and M. Andreani , “Identification of the Novel Allele HLA‐DQB1*06:02:61 by Next‐Generation Sequencing,” HLA 104, no. 2 (August 2024): e15636, 10.1111/tan.15636. 39101361

[94] V. Oliveira , J. Pinto , R. Vianna , L. C. Porto , and D. Secco , “Characterisation of the Novel HLA‐DQB1*06:02:62 Allele Using Short‐ and Long‐Read Sequencing Technologies,” HLA 104, no. 2 (August 2024): e15663, 10.1111/tan.15663. 39189304

[95] I. F. Molinari , A. M. L. de Oliveira , F. P. Massi , Q. A. de Lima Neto , and J. E. L. Visentainer , “The Novel HLA Allele, HLA‐DQB1*02:211, Identified in a Brazilian Bone Marrow Donor,” HLA 104, no. 2 (August 2024): e15642, 10.1111/tan.15642. 39132708

[96] L. A. Marin Rubio and J. Ontanon , “Eight New Variants of HLA‐DQB1*03 Identified by Next‐Generation Sequencing,” HLA 104, no. 2 (August 2024): e15634, 10.1111/tan.15634. 39091246

[97] D. Kouniaki , V. Kitsiou , and A. Tsirogianni , “A Novel HLA‐DQB1*03 Variant Allele, HLA‐DQB1*03:01:01:64, Identified by Next‐Generation Sequencing,” HLA 104, no. 3 (September 2024): e15705, 10.1111/tan.15705. 39311754

[98] D. Secco , V. Oliveira , M. Cunha , R. Vianna , and L. C. Porto , “Characterisation of the Novel HLA‐DQB1*03:01:61 Allele, First Identified in a Brazilian Individual,” HLA 103, no. 6 (June 2024): e15589, 10.1111/tan.15589. 38934065

[99] D. AlAbduladheem , S. AlZahrani , M. Alnajjar , M. AlAithan , and M. Awaji , “Identification of the Novel Allele, HLA‐DQB1*03:517, in a Saudi Individual,” HLA 104, no. 2 (August 2024): e15624, 10.1111/tan.15624. 39091248

[100] L. A. Marin Rubio and J. Ontanon , “Characterisation of Five New Variants of HLA‐DPA1,” HLA 103, no. 6 (June 2024): e15580, 10.1111/tan.15580. 38932664

[101] M. Cargou , M. Andreani , M. Battarra , M. Ralazamahaleo , and J. Visentin , “Characterisation of the Novel HLA‐DPA1*01:12:03 Allele by Sequencing‐Based Typing,” HLA 104, no. 3 (September 2024): e15674, 10.1111/tan.15674. 39234795

[102] M. R. Moya‐Quiles and M. Muro , “A Novel HLA‐DPA1 Allele, HLA‐DPA1*02:134, Identified by Next‐Generation Sequencing,” HLA 104, no. 1 (July 2024): e15603, 10.1111/tan.15603. 39022866

[103] M. R. Moya‐Quiles and M. Muro , “Characterisation of Two New HLA‐DPA1 Alleles: HLA‐DPA1*02:139 and ‐DPA1*02:140N,” HLA 104, no. 3 (September 2024): e15696, 10.1111/tan.15696. 39291370

[104] E. Khamaganova , E. Leonov , I. Pavlova , A. J. E. French , and S. J. F. Hopper , “The Novel HLA‐DPB1*04:02:01:44 Allele Identified in a Volunteer Bone Marrow Donor,” HLA 104, no. 2 (August 2024): e15659, 10.1111/tan.15659. 39171357

[105] F. Wang , C. Chen , N. Chen , W. Zhang , and F. Zhu , “Characterisation of the Novel HLA‐DPB1*04:02:24 Allele by Next‐Generation Sequencing,” HLA 104, no. 3 (September 2024): e15682, 10.1111/tan.15682. 39279439

[106] A. M. L. de Oliveira , I. F. Molinari , F. P. Massi , L. D. B. Pinto , and J. E. L. Visentainer , “Identification of the Novel HLA‐DPB1*14:01:15 Allele in a Brazilian Bone Marrow Donor,” HLA 104, no. 2 (August 2024): e15637, 10.1111/tan.15637. 39091266

[107] W. Zhang , N. Chen , W. Pi , F. Wang , and F. Zhu , “Identification of the Novel HLA‐DPB1*21:01:02 Allele Using Nanopore Sequencing Technology,” HLA 104, no. 3 (September 2024): e15666, 10.1111/tan.15666. 39219378

[108] B. B. Gao , X. F. Xia , L. An , H. C. Xue , and C. T. Zhang , “The Novel HLA‐DPB1*1553:01 Allele, Identified by Sanger Dideoxy Nucleotide Sequencing in a Chinese Individual,” HLA 103, no. 6 (June 2024): e15510, 10.1111/tan.15510. 38887192

[109] M. Cargou , V. Elsermans , I. Top , E. Wojciechowski , and J. Visentin , “Characterisation of the Novel HLA‐DPB1*1618:01 Allele by Sequencing‐Based Typing,” HLA 104, no. 3 (September 2024): e15678, 10.1111/tan.15678. 39234817

[110] M. Ivanova and V. Shivarov , “Characterisation of the Novel MICA*112:02 Allele by Next Generation Sequencing,” HLA 103, no. 6 (June 2024): e15583, 10.1111/tan.15583. 38923360

[111] Z. R. Quan , H. Y. Zou , J. Liu , B. N. Yang , and J. M. Song , “MICB*002:06, a Novel Exonic Variant of MICB (MHC Class I Chain‐Related Gene B),” HLA 104, no. 2 (August 2024): e15639, 10.1111/tan.15639. 39132702

[112] V. Shivarov and M. Ivanova , “Characterisation of Novel MICB Alleles by Next Generation Sequencing: MICB*055 and MICB*005:15,” HLA 103, no. 6 (June 2024): e15581, 10.1111/tan.15581. 38923355

